# Treatment variability for shoulder pain between physician and non-physician clinicians based on initial setting and specific shoulder diagnosis: a health system analysis

**DOI:** 10.1186/s12913-025-13175-w

**Published:** 2025-10-16

**Authors:** Daniel I. Rhon, Maggie E. Horn, Hui-Jie Lee, Sarah Morton-Oswald, Steven Z. George

**Affiliations:** 1 Department of Rehabilitation Medicine, Uniformed Services University, 4301 Jones Bridge Road, Bethesda, MD USA; 2 Department of Rehabilitation Medicine, Brooke Army Medical Center, 3551 Roger Brooke Drive, San Antonio, TX USA; 3 Departments of Orthopaedic Surgery and Population Health Sciences, School of Medicine, Duke University, 200 Morris Street, Durham, NC USA; 4 Department of Biostatistics and Bioinformatics, Duke University School of Medicine, 2424 Erwin Rd, Durham, NC USA; 5 Duke Clinical Research Institute, School of Medicine, Duke University, 300 Morgan Street, Durham, NC USA

**Keywords:** Shoulder injury, Shoulder pain, Health system, Military medicine, Physician, Non-physician, Care pathways

## Abstract

**Background:**

Shoulder pain is common, can arise from various causes and has a highly variable prognosis. Treatment may differ based on the clinician delivering the care and initial care settings (primary, specialty, or emergency care). The purpose of this study was to investigate how the management of shoulder pain differs depending on clinical care settings and clinician type.

**Methods:**

This was an observational cohort study. Using routinely collected health information from Military Health System electronic medical records and claims data, we assessed initial care setting and provider types for common shoulder disorders that occurred between July 1, 2013, and March 31, 2019. We identified shoulder-related care using current procedural terminology (CPT) and ICD-10 diagnosis codes marked in encounters within three months of initial diagnosis. Care was categorized into pharmacological treatment, non-pharmacological treatment, and imaging procedures, and compared across initial care settings and provider types.

**Results:**

There were 246,041 unique individuals in the cohort with a mean(SD) age of 37.9(12.3) years, 21.9% female, 63.1% on active duty, and 76.3% enlisted. Most patients were initially seen in a primary care setting (80.2%), followed by specialty care (16.7%) and emergency care(3.1%), and 44.4% of the patients were seen by physicians. Across all settings and clinician types, non-specific shoulder diagnosis (i.e. non-specific or unspecified shoulder pain) was the most common (73.9%), followed by rotator cuff-related pain disorders (15.9%), multiple specific diagnoses (3.7%), glenohumeral osteoarthritis (2.0%), and hypomobility disorders (1.8%). Patients who saw a specialty care non-physician were more likely to receive exercise or physical therapy than other clinician-location types, which was fairly consistent across all diagnostic groups.

**Conclusion:**

Diagnostic subgroups of shoulder pain and initial care settings influence practice variability between physician and non-physician management of shoulder pain. Additionally, three out of every 4 patients received a non-specific shoulder diagnosis. These findings highlight the lack of coding using specific diagnostic labels, which prevents a deeper assessment of care care variability across specific shoulder diagnostic subgroups. Initial care settings should also be considered when exploring variability in care pathways for shoulder disorders.

**Supplementary Information:**

The online version contains supplementary material available at 10.1186/s12913-025-13175-w.

## Introduction

Shoulder disorders are the third most common musculoskeletal pain complaints seen in primary care [1]. Both the volume of and the burden on the health system have increased over time [2]. Shoulder disorders vary substantially in etiology and prognosis, leading to a variety of different treatment approaches. Patient, provider, and system-level factors can influence the long-term trajectory and outcomes related to shoulder disorders [3, 4]. 

Treatment patterns for the same condition can vary depending on the clinician’s profession and healthcare setting. While most shoulder disorders are initially seen by primary care clinicians, patients can also be seen in specialty or emergency care clinics [5, 6], by either physicians or licensed non-physician clinicians (i.e., physician assistant, nurse practitioner, or physical therapist). Variable adherence to clinical practice guidelines for low back pain was seen across physicians in general practice, emergency care, and occupational care specialties [7]. The type of provider a patient initially consults with can influence downstream healthcare utilization and outcomes for other musculoskeletal disorders [5, 8]. For example, a scoping review found variability in five downstream healthcare utilization outcomes (medication, imaging, care seeking, cost, and procedures) depending on the initial clinician seen for low back pain (physician vs. non-physician) [5]. Similar results were found in patients with neck pain [9]. Physicians and non-physicians have equal performance in managing chronic disease [10], diabetes-specific outcomes [11], and quality of medication prescription [12], but with conflicting reports on differences in frequency and appropriateness of imaging orders [13–15]. No similar work has been conducted for management of shoulder conditions.

Health systems are looking to improve patient-centered care by increasing patient satisfaction, optimizing outcomes, and maximizing value [16]. This becomes challenging while also managing a large physician shortage [17]. This requires optimizing access to care with the use of non-physician clinicians. Understanding the variability across clinical care settings (primary care, specialty care, and emergency care) is necessary to improve value-based care, especially as patients often cross settings over their course of care. Currently, the exploration of the effects of these synergies on health system outcomes and efficiency are largely unexplored.

The purpose of this study was to compare differences in management for common shoulder disorders based on initial care setting (primary, emergency, or specialty care) and initial clinician managment (physician or non-physician). Our hypothesis was that non-physician clinicians would see a higher proportion of patients and that patients initially seen in emergency and/or specialty care settings would have higher rates of healthcare utilization. We also expected exercise utilization to be higher than pharmacological treatment, across all sites and for all diagnostic subgroups. These results will help establish future healthcare policy and allow for comparisons to such findings in other musculoskeletal disorders [18–20]. 

## Methods

This cohort study used routinely collected health information to identify healthcare patterns from the initial shoulder care visit prospectively for a 3-month period. The study received ethics approval from the Institutional Review Board at Brooke Army Medical Center. It was deemed exempt due to use of de-identified data and therefore the consent requirement was waived. The REporting of studies Conducted using Observational Routinely-collected Data (RECORD) [21] extension of the Strengthening the Reporting of OBservational studies in Epidemiology (STROBE) checklist was used to guide reporting.

### Setting and participants

Participants were adults 18 to 65 years of age (17 years if emancipated minors on active duty), seeking care for shoulder pain between July 2013 and March 2019. All patients were beneficiaries of the Military Health System (MHS), one of the largest health systems in the United States (45 hospitals and 566 ambulatory clinics supporting nearly 10 million beneficiaries) [22]. Participants were eligible if they had at least one visit for a shoulder-related disorder in an outpatient military treatment facility and seen by a credentialed clinician. Their initial visit was termed the index date. Six months of eligibility prior to the index date and three months of eligibility after the index date were also required. Only individuals with no shoulder care in the prior six months were included. To improve the homogeneity of the sample, we excluded individuals with shoulder fractures, trauma, or open dislocations, as well as anyone with an upper extremity amputation. We excluded patients who did not have their index visits in a military clinic because claims data for the private sector do not reliably capture associated provider type and clinic setting. Patients with missing index provider type or clinic setting, or those who were only seen by non-clinicians (e.g. nurses or technicians), or visited multiple settings on the index date (e.g. primary care and emergency care) were further excluded from the analysis.

### Data source

Data were sourced from the MHS Data Repository (MDR), which houses person-level electronic medical record (EMR) data and claims data for all outpatient encounters in both military and private section civilian clinics where beneficiaries seek care. There are over 250 unique data sources that feed into the MDR and missing data are continuously cross-referenced across multiple data sources for validation.

### Variables

#### Shoulder care

We identified patients based on encounters coded with a shoulder-related International Statistical Classification of Diseases and Related Health Problems (ICD) code 9th and 10th edition. We categorized all shoulder care within 3 months of the index date based on ICD codes subgrouped by rotator cuff (RC) related pain syndromes, acromioclavicular (AC) joint pain and dysfunction, glenohumeral instability (to include closed dislocations and subluxations), glenohumeral hypomobility (to include adhesive capsulitis), glenohumeral osteoarthritis, and non-specific shoulder pain. Patients with a non-specific shoulder diagnosis and a specific diagnosis were put into the corresponding specific diagnosis category. Finally, we placed individuals with multiple specific diagnoses into a caterogry of more than one shoulder diagnosis. We characterized shoulder-related care using Current Procedural Terminology (CPT) codes linked to shoulder-related encounters. For all follow-up care we included encounters from both military and private sector clinics. We also included radiology and prescription data using American Hospital Formulary Service (AHFS) therapeutic class codes, all sourced from the MDR. The list of codes can be found in the appendix (Tables SA.8-SA.13).

#### Clinician type and setting

No current standard exists for classifying clinicians types, with multiple approaches used in different scenarios. Some classification systems use unique proprietary markers [23], while others use health provider taxonomy codes [24, 25]. However, the assigned classification does not guarantee the provider works in the corresponding clinical care setting [26], which is why we chose to focus more on clinic types, where actual care took place. The majority of clinical care pathways for managing musculoskeletal pain endorse a stepped care approach, where care should initiate in primary care and only escalate to specialty providers if symptoms persist or are unresponsive to initial lines of care. We categorized clinicians delivering the index visit shoulder-related care using the SKILL variable from MDR (1 = physician; 2 = non-physician). Patients seeing both physicians and non-physicians on the index date were classified as seen by physicians. We excluded cases where a patient did not see a clinician on the index visit (SKILL ≠ 1 or 2). We used product line (PRODLINE) and Medical Expense and Performance Reporting System (MEPRS) codes to identify the settings where initial care took place, as either primary care, emergency care, or specialty care (i.e., orthopaedics, physical medicine, physical therapy). We excluded cases where a patient visited multiple locations on the index date.

#### Outcome variables

We assessed healthcare utilization that occurred within the first three months after the index shoulder visit, broadly classified into three main groups: pharmacological treatments, non-pharmacological treatments, and imaging. The number of prescriptions and treatments per patient. and a binary indicator of specific treatment receipt were calculated. Pharmacological treatments included NSAIDS, opioids, benzodiazepines, muscle relaxers, analgesics, and steroid joint injections. Non-pharmacological treatments included physical therapy (anyone seen by a physical therapist), and other specific interventions regardless of who delivered them: exercise therapy, passive modalities (e.g., electrical stimulation, hot/cold therapy), manual therapy, and acupuncture. Imaging studies included radiographs, magnetic resonance imaging (MRI), arthrograms, and computed tomography (CT) scans. Imaging was also further categorized as plain radiographs or advanced imaging, the latter of which was the combination of MRIs, arthrograms, or CT scans. We also determined the timing of imaging and classified patients as having received advanced imaging only, radiographs only, or a combination of the two (and in which order). It’s important to note that all clinicians working in this health system have priveleges for ordering radiographs and advanced imaging (e.g., occupational and physical therapists).

### Statistical analysis

Diagnostic subgroups were designated based on clinician and setting seen for their initial shoulder encounter. We summarized healthcare utilization for each shoulder disorder subgroup and compared utilization events across type of clinician and settings (primary care, emergency care, or specialty care). Descriptive statistics, including mean and standard deviations, summarized the number of times the care was received per patient. Given the large sample size of this study, a small non-clinically important difference can be claimed as statistically significant with a *p*-value < 0.05 [27]. Therefore, we prioritized the focus on clinical relevance and did not perform inferential statistical testing.

## Results

There were 246,041 individuals meeting inclusion criteria (Table 1), with a mean (SD) age of 37.9 (12.3) years, 21.9% female, 63.1% on active duty, and 76.3% enlisted (or with enlisted sponsor; Table 2). A non-specific shoulder disorder diagnosis was the most common (73.9% of all cases). These individuals never received a more specific diagnosis. Rotator cuff related pain disorders (15.9%), glenohumeral osteoarthritis (2.0%), and hypomobility disorders (1.8%) were the next most common. Patients with more than one diagnosis accounted for 3.7% of the cohort. Patients with a non-specific diagnosis had the fewest mean [SD] visits per patient (2.8 [3.3]) and patients with multiple diagnoses and hypomobility disorders had the highest (8.3[5.7] and 6.8[5.8], respectively). Full demographic data based on disease group is shown in Table 2.

**Table 1:** Derivation of final cohort

	**Inclusion/Exclusion Criteria**	**Included**	**Excluded**
**1**	Sought care for shoulder pain in the MHS from January 2013 to July 2019	1,488,821	---
**2**	Had care for shoulder pain in the year prior to index date	1,285,500	203,321
3	Individuals excluded for being younger than 18 or older than 65 years	1,156,582	128,918
**4**	Did not have the minimum eligibility periods	663,573	493,009
Final value from Automated MDR data extraction above. Below are results from further manual validation of data received			
**5**	Had at least one shoulder-related diagnosis or procedure code in CAPER, TEDNI, Ancillary, TEDI, or SIDR from January 2013 to July 2019	586,871	---
**6**	Had an index date (first shoulder-related diagnosis or procedure) in an outpatient setting (CAPER or TEDNI) from July 2013 to March 2019. This allows for 6 months of pre-index washout period and 3 months of post-index follow-up.	564,825	22,046
**7**	Had MHS eligibility from 6 months before index date to 3 months after index date, allowing a 6-month gap in eligibility	563,239	1,586
**8**	Had no evidence of trauma from the index date to 3 months after	460,562	102,677
**9**	Had no evidence of open dislocation from the index date to 3 months after	460,544	18
**10**	Had no evidence of shoulder/humerus fracture from index date to 3 months after	456,449	4,095
**11**	Had no evidence of an amputation of the upper extremity not including only the digits from the index date to 3 months after	456,440	9
**12**	17-65 years old on the index date	456,241	199
**13**	Had an index visit from a military clinic	262,604	193,637
**14**	Had non-missing index clinician types and settings	260,176	2,428
**15**	Had an index visit with a clinician	247,293	12,883
**16**	Did not visit multiple settings on the index date	246,041	1,252

*MHS* Military Health System, *MDR* Military Health System Data Repository, *CAPER* Comprehensive Ambulatory Professional Encounter Record, *TEDNI* TRICARE Encounter Data – Not Institutional, *SIDR* Standard Inpatient Data Record, *TEDI* TRICARE Encounter Data – Institutional

**Fig. 1:**
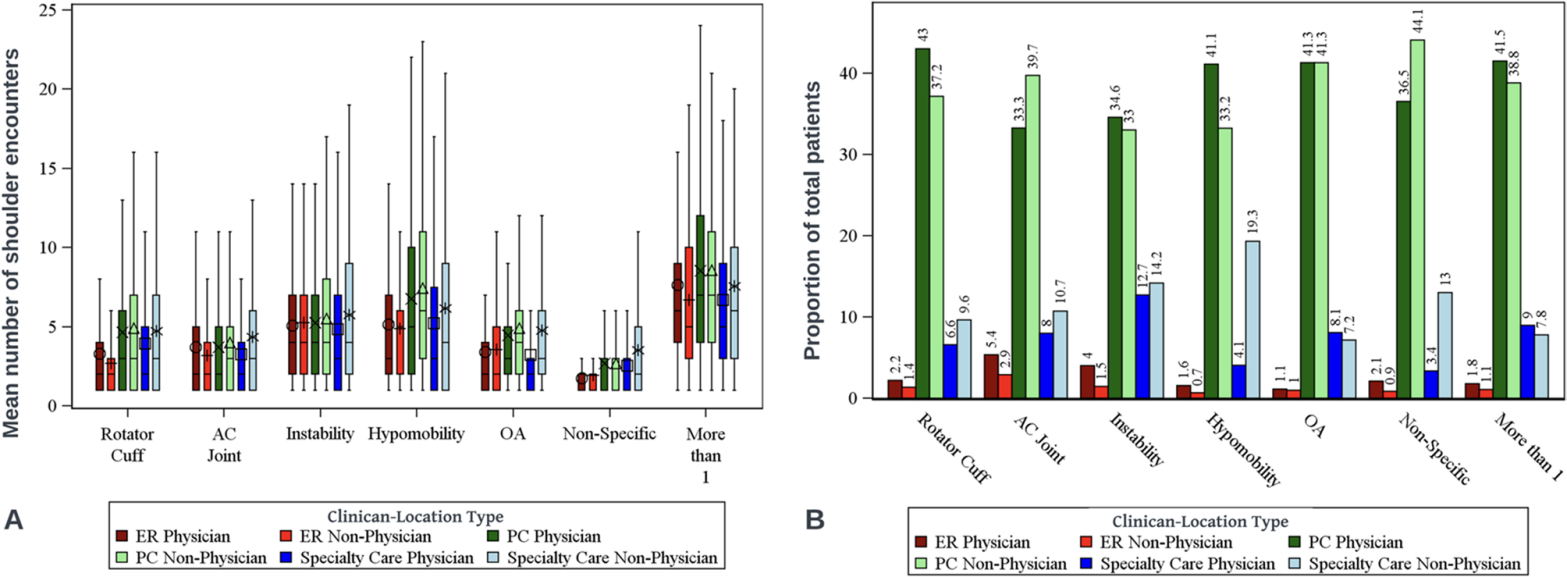
Proportion of patients and mean encounters by shoulder diagnosis, setting, and clinician type. Note: A = Mean number of shoulder encounters. B = Proportion of patients seen by each provider type. AC = Acromioclavicular; OA = Osteoarthritis. Data to inform the figures is presented in supplementary Tables SA1 to SA7

All results reflect categorization based on the setting or clinician type that initially managed each patient on their index date. Across the entire cohort, without consideration for diagnostic subgroup, non-physician clinicians saw 55.6% (136,749) and physicians saw 44.4% (109,292) of all patients (Table 2). However, variatons emerged across settings and within diagnostic subgroups. In emergency care settings, physicians saw more than double the caseload of non-physicians (68.7% versus 31.3%), but in specialty care the workload of physicians was approximately a quarter that of non-physicians (26.4% versus 73.6%). Of the specific single diagnosis groups, hypomobility and AC joint dysfunction were proportionately seen more often by non-physicians (Supplement Figure SA1 and Table 2). The non-specific shoulder pain group was seen more often by non-physicians than physicians (58% versus 42%; Table 2). The mean number of visits per patient was highest (6.78) for hypomobility and lowest (2.78) for non-specific shoulder disorders (Fig. 1; Table 2 and Supplement Figure SA2). Those with more than one diagnoses had the highest mean number of visits (8.27).

**Table 2:** Patient characteristics by disease group

	Rotator Cuff (*N*=39030)	AC Joint (*N*=2237)	Instability/Dislocation (*N*=4300)	Hypomobility/Adhesive Capsulitis (N=4528)	Osteoarthrosis (*N*=4910)	Non-Specific Diagnosis (*N*=181946)	More than 1 Specific Disease (*N*=9090)	Total (*N*=246041)
**Index Clinician Type**								
Physician	20224 (51.8%)	1043 (46.6%)	2207 (51.3%)	2117 (46.8%)	2481 (50.5%)	76467 (42.0%)	4753 (52.3%)	109292 (44.4%)
Non-Physician	18806 (48.2%)	1194 (53.4%)	2093 (48.7%)	2411 (53.2%)	2429 (49.5%)	105479 (58.0%)	4337 (47.7%)	136749 (55.6%)
**Index Location Type**								
Emergency Room	1397 (3.6%)	185 (8.3%)	236 (5.5%)	102 (2.3%)	105 (2.1%)	5448 (3.0%)	263 (2.9%)	7736 (3.1%)
Primary Care	31292 (80.2%)	1633 (73.0%)	2907 (67.6%)	3367 (74.4%)	4056 (82.6%)	146686 (80.6%)	7301 (80.3%)	197242 (80.2%)
Specialty Care	6341 (16.2%)	419 (18.7%)	1157 (26.9%)	1059 (23.4%)	749 (15.3%)	29812 (16.4%)	1526 (16.8%)	41063 (16.7%)
**Index Clinician Location**								
ER Physician	862 (2.2%)	120 (5.4%)	173 (4.0%)	71 (1.6%)	56 (1.1%)	3865 (2.1%)	164 (1.8%)	5311 (2.2%)
ER Non-Physician	535 (1.4%)	65 (2.9%)	63 (1.5%)	31 (0.7%)	49 (1.0%)	1583 (0.9%)	99 (1.1%)	2425 (1.0%)
PC Physician	16787 (43.0%)	744 (33.3%)	1487 (34.6%)	1862 (41.1%)	2028 (41.3%)	66471 (36.5%)	3773 (41.5%)	93152 (37.9%)
PC Non-Physician	14505 (37.2%)	889 (39.7%)	1420 (33.0%)	1505 (33.2%)	2028 (41.3%)	80215 (44.1%)	3528 (38.8%)	104090 (42.3%)
Specialty Care Physician	2575 (6.6%)	179 (8.0%)	547 (12.7%)	184 (4.1%)	397 (8.1%)	6131 (3.4%)	816 (9.0%)	10829 (4.4%)
Specialty Care Non-Physician	3766 (9.6%)	240 (10.7%)	610 (14.2%)	875 (19.3%)	352 (7.2%)	23681 (13.0%)	710 (7.8%)	30234 (12.3%)
**Patient Sex**								
Missing	8540 (21.9%)	328 (14.7%)	989 (23.0%)	1056 (23.3%)	1007 (20.5%)	47870 (26.3%)	2013 (22.1%)	61803 (25.1%)
Female	8985 (23.0%)	261 (11.7%)	550 (12.8%)	1674 (37.0%)	880 (17.9%)	39701 (21.8%)	1892 (20.8%)	53943 (21.9%)
Male	21505 (55.1%)	1648 (73.7%)	2761 (64.2%)	1798 (39.7%)	3023 (61.6%)	94375 (51.9%)	5185 (57.0%)	130295 (53.0%)
**Patient Age**								
Mean (SD)	39.80 (12.10)	30.68 (9.68)	28.64 (8.63)	46.32 (11.95)	46.39 (10.98)	36.98 (12.11)	44.62 (11.83)	37.87 (12.31)
Median	39.0	28.0	26.0	48.0	47.0	35.0	45.0	36.0
Q1, Q3	30.0, 49.0	23.0, 36.0	22.0, 33.0	38.0, 56.0	38.0, 56.0	27.0, 46.0	36.0, 54.0	27.0, 47.0
Range	(17.0-65.0)	(17.0-65.0)	(18.0-65.0)	(18.0-65.0)	(19.0-65.0)	(18.0-65.0)	(18.0-65.0)	(17.0-65.0)
**Index Date Year**								
2013	0 (0.0%)	0 (0.0%)	0 (0.0%)	0 (0.0%)	88 (1.8%)	0 (0.0%)	0 (0.0%)	88 (0.0%)
2014	4956 (12.7%)	397 (17.7%)	280 (6.5%)	651 (14.4%)	828 (16.9%)	16599 (9.1%)	1386 (15.2%)	25097 (10.2%)
2015	6924 (17.7%)	716 (32.0%)	703 (16.3%)	711 (15.7%)	800 (16.3%)	25298 (13.9%)	1442 (15.9%)	36594 (14.9%)
2016	10274 (26.3%)	547 (24.5%)	1269 (29.5%)	1134 (25.0%)	1278 (26.0%)	46948 (25.8%)	2376 (26.1%)	63826 (25.9%)
2017	8149 (20.9%)	315 (14.1%)	1042 (24.2%)	953 (21.0%)	928 (18.9%)	43727 (24.0%)	1778 (19.6%)	56892 (23.1%)
2018	7168 (18.4%)	209 (9.3%)	818 (19.0%)	848 (18.7%)	789 (16.1%)	39129 (21.5%)	1681 (18.5%)	50642 (20.6%)
2019	1559 (4.0%)	53 (2.4%)	188 (4.4%)	231 (5.1%)	199 (4.1%)	10245 (5.6%)	427 (4.7%)	12902 (5.2%)
**Patient Beneficiary Category**								
Missing	206 (0.5%)	11 (0.5%)	20 (0.5%)	32 (0.7%)	23 (0.5%)	946 (0.5%)	30 (0.3%)	1268 (0.5%)
Dependent	7388 (18.9%)	215 (9.6%)	412 (9.6%)	1491 (32.9%)	756 (15.4%)	32783 (18.0%)	1647 (18.1%)	44692 (18.2%)
Retired Service Member	6270 (16.1%)	102 (4.6%)	109 (2.5%)	1065 (23.5%)	1399 (28.5%)	21618 (11.9%)	2386 (26.2%)	32949 (13.4%)
Other	1724 (4.4%)	31 (1.4%)	85 (2.0%)	358 (7.9%)	222 (4.5%)	8438 (4.6%)	501 (5.5%)	11359 (4.6%)
Active Duty	23328 (59.8%)	1874 (83.8%)	3659 (85.1%)	1574 (34.8%)	2493 (50.8%)	117799 (64.7%)	4509 (49.6%)	155236 (63.1%)
Inactive Guard/Reserve	114 (0.3%)	4 (0.2%)	15 (0.3%)	8 (0.2%)	17 (0.3%)	362 (0.2%)	17 (0.2%)	537 (0.2%)
**Pay Plan**								
Missing	208 (0.5%)	11 (0.5%)	20 (0.5%)	33 (0.7%)	23 (0.5%)	952 (0.5%)	30 (0.3%)	1277 (0.5%)
Other/Unknown	121 (0.3%)	1 (0.0%)	0 (0.0%)	10 (0.2%)	13 (0.3%)	445 (0.2%)	24 (0.3%)	614 (0.2%)
Cadet	135 (0.3%)	63 (2.8%)	201 (4.7%)	3 (0.1%)	2 (0.0%)	842 (0.5%)	38 (0.4%)	1284 (0.5%)
Enlisted	28982 (74.3%)	1775 (79.3%)	3286 (76.4%)	3141 (69.4%)	3439 (70.0%)	140630 (77.3%)	6504 (71.6%)	187757 (76.3%)
Officer	9584 (24.6%)	387 (17.3%)	793 (18.4%)	1341 (29.6%)	1433 (29.2%)	39077 (21.5%)	2494 (27.4%)	55109 (22.4%)
**Service**								
Missing	207 (0.5%)	11 (0.5%)	20 (0.5%)	32 (0.7%)	23 (0.5%)	950 (0.5%)	30 (0.3%)	1273 (0.5%)
Other/Unknown	122 (0.3%)	2 (0.1%)	6 (0.1%)	41 (0.9%)	19 (0.4%)	436 (0.2%)	37 (0.4%)	663 (0.3%)
Army	15511 (39.7%)	895 (40.0%)	1661 (38.6%)	1746 (38.6%)	2377 (48.4%)	80284 (44.1%)	3911 (43.0%)	106385 (43.2%)
Coast Guard	590 (1.5%)	35 (1.6%)	37 (0.9%)	53 (1.2%)	53 (1.1%)	1698 (0.9%)	151 (1.7%)	2617 (1.1%)
Air Force	10298 (26.4%)	463 (20.7%)	821 (19.1%)	1292 (28.5%)	1214 (24.7%)	47501 (26.1%)	2359 (26.0%)	63948 (26.0%)
Marines	3996 (10.2%)	376 (16.8%)	799 (18.6%)	347 (7.7%)	383 (7.8%)	17826 (9.8%)	820 (9.0%)	24547 (10.0%)
Navy	8306 (21.3%)	455 (20.3%)	956 (22.2%)	1017 (22.5%)	841 (17.1%)	33251 (18.3%)	1782 (19.6%)	46608 (18.9%)
**Shoulder care encounters within 3 months**								
Mean (SD)	4.65 (4.52)	3.85 (3.84)	5.35 (4.73)	6.78 (5.78)	4.55 (3.95)	2.78 (3.25)	8.27 (5.61)	3.44 (3.92)
Median	3.0	2.0	4.0	5.0	3.0	1.0	7.0	2.0
Q1, Q3	1.0, 6.0	1.0, 5.0	2.0, 8.0	2.0, 10.0	2.0, 6.0	1.0, 3.0	4.0, 11.0	1.0, 4.0
Range	(1.0-42.0)	(1.0-32.0)	(1.0-50.0)	(1.0-43.0)	(1.0-29.0)	(1.0-39.0)	(1.0-40.0)	(1.0-50.0)

Most patients were seen primary care settings (80.2%), followed by specialty care (16.7%), and then emergency care (3.1%). This trend was consistent across the diagnostic subgroups. The highest proportion of patients seen in specialty care clinics were patients with instability and hypomobility (26.9% and 23.4% respectively of all cases in this diagnostic subgroup; Table 2 and Supplement Tables SA3 and SA4) and the lowest proportion in specialty care was among patients with osteoarthritis (15.3%) and rotator cuff disorders (16.2%; Table 2). The highest proportion of patients seen in emergency care settings was AC joint at 8.3% (Table 2) and the lowest was osteoarthritis at 2.1% (Supplementary Table SA5) of all cases (Table 2). In primary care, the largest proportion was osteoarthritis (82.6%) and non-specific shoulder pain (80.6%; Table 2 and Supplement Figure SA3).

### Pharmacological treatment

**Fig. 2:**
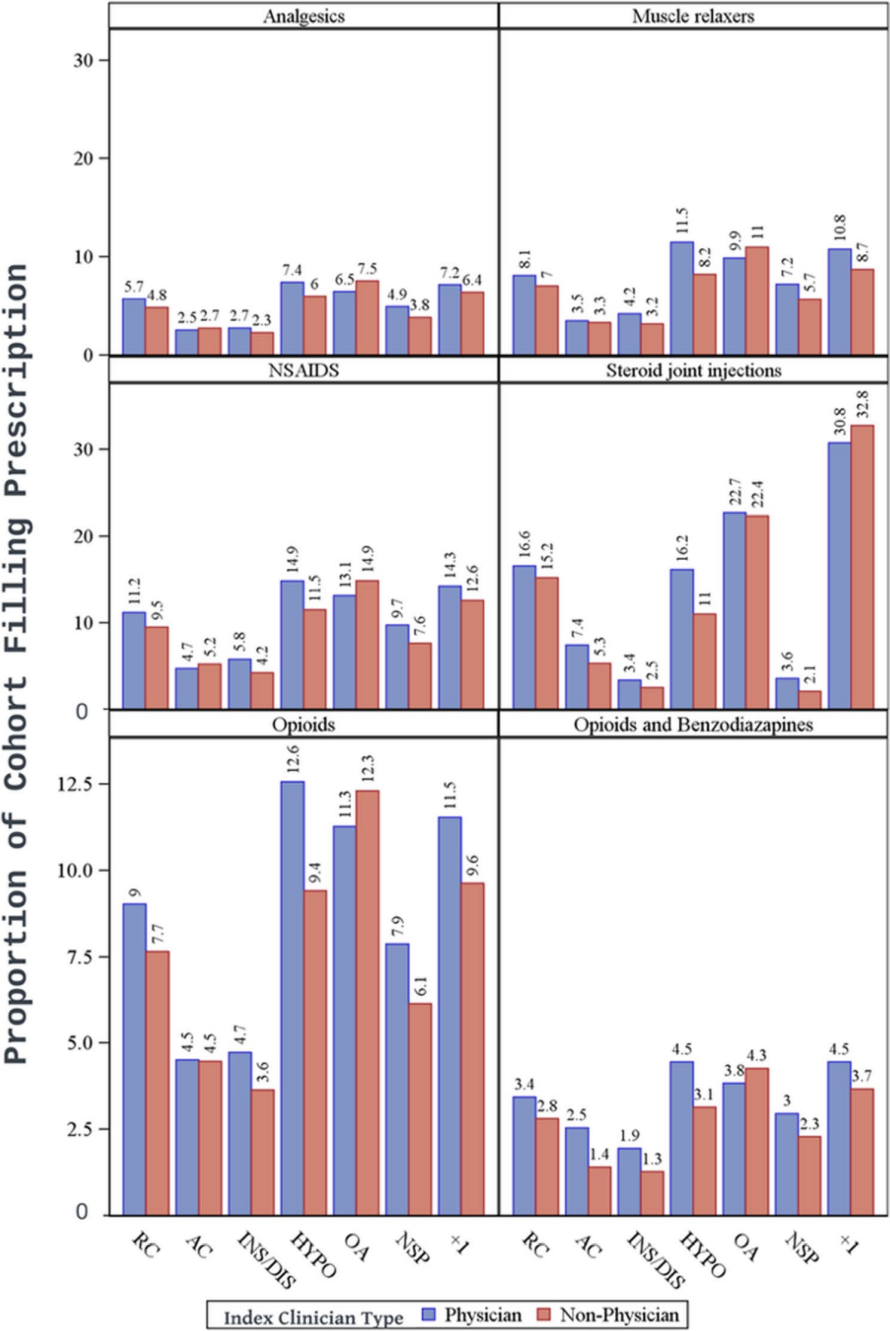
Medication prescription rates are based on index clinician type for each shoulder diagnostic category. Note: RC = Rotator Cuff; AC= Acromioclavicular; INS/DIS = instability/dislocation; HYPO = hypomobility (including adhesive capsulitis); OA = Osteoarthritis; NSP = non-specific shoulder pain; +1 = more than 1 specific shoulder diagnostic category

In general, use of pharmacological treatment was highest in patients with osteoarthritis, rotator cuff, hypomobility, and those with more than one specific shoulder disorder group (Supplemental Tables SA1, SA4, SA5, SA7, and Figure SA3). Corticosteroid injection was the most common pharmacological treatment in general for all shoulder disorder groups except for the instability and non-specific shoulder groups (Fig. 2). Pharmacological treatment was used the least for patients with glenohumeral instability. Without consideration for settings, prescriptions were similar across most diagnostic subgroups, but marginally more likely for patients initially seen by a physician versus non-physician (Fig. 2). Opioid prescriptions were used the most for osteoarthritis and hypomobility (11.9% and 11.0% of all patients, respectively) and the least for instability (4.3%). However, variations in prescribing patterns emerged across different clinical care settings, revealing some notable exceptions. For osteoarthriritis, all medication types in all settings were prescribed more frequently by non-physicians than physicians (except for corticosteroid injections in specialty care, 36.9% versus 39.0%). For AC joint dysfunction, there were higher NSAID prescription rates by non-physicians than physicians in emergency (9.2% versus 5.0%), but relatively equal in primary care settings (5.7% versus 5.2%). Also relatively equal between provider types in this setting for other analgesics (3.3% versus 2.8%) and opioids (4.9% versus 4.8%). Corticosteroid injections were more commonly prescribed by non-physicians than physicians for instability (4.8% versus 2.9%) and hypomobility (19.4% versus 9.9%) in emergency care settings, but relatively equal for patients with more than one shoulder diagnosis in primary care (32.6% versus 31.8%) and specialty care (36.2% versus 29.3%; supplement Figure SA.3).

### Non-pharmacological treatment: (32.1% for non-specific – 33.6% for OA

**Fig. 3:**
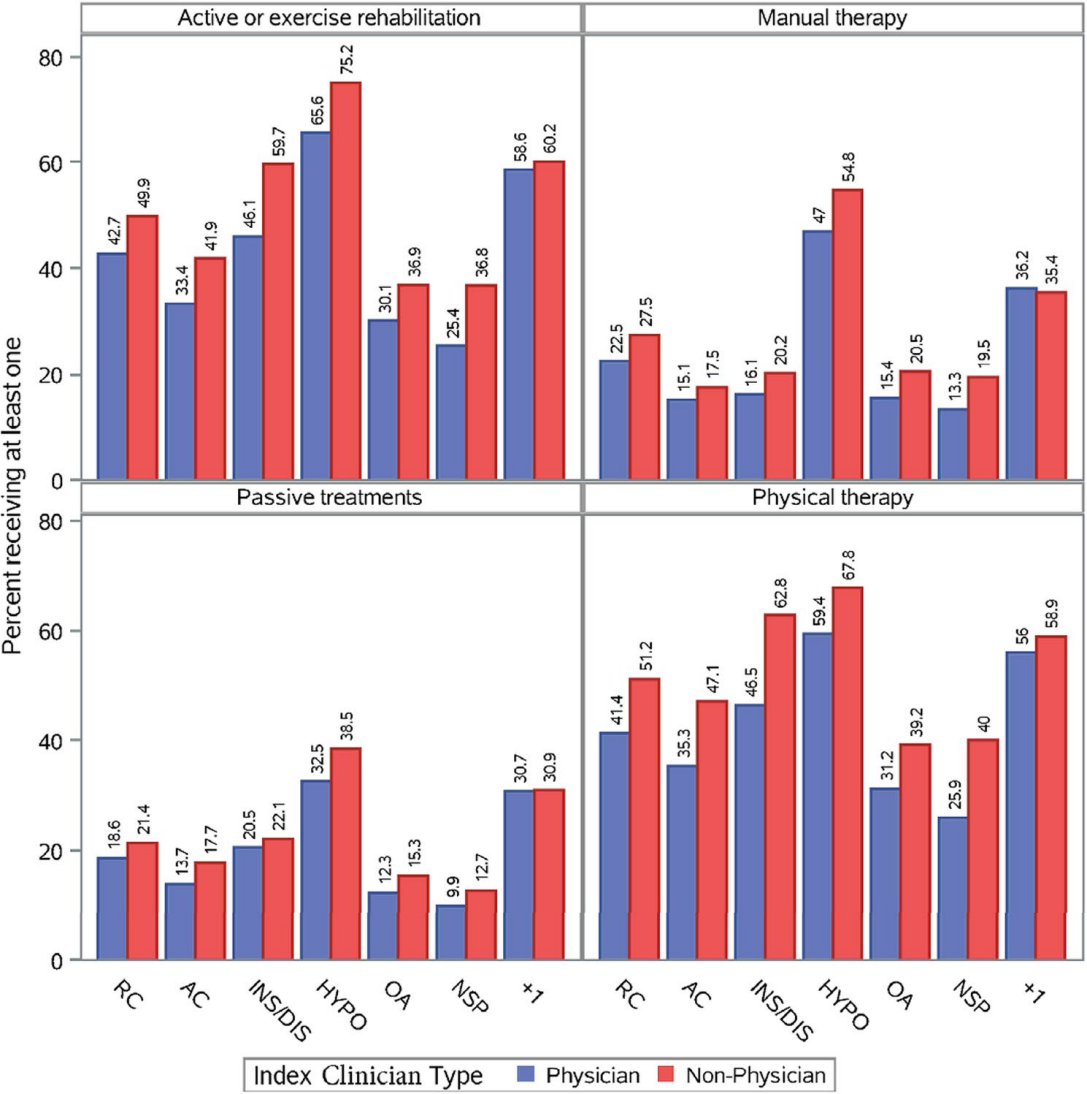
Non-pharmacological treatment use based on index clinician type for each shoulder diagnostic category. Note: RC = Rotator Cuff; AC = Acromioclavicular; INS/DIS = instability/dislocation; HYPO = hypomobility (including adhesive capsulitis); OA = Osteoarthritis; NSP = non-specific shoulder pain; +1 = more than 1 specific shoulder diagnostic category

In general, non-pharmacological treatment was more common than pharmacological. Patients were marginally more likely to receive at least one type of non-pharmacological treatment if they initially saw a non-physician across almost every diagnostic category, without accounting for setting (Fig. 3). Across the board, a substantial portion of patients received exercise and physical therapy, ranging from 32.1% to 34.3% of patients with non-specific shoulder disorders to 71.1% and 64.3% of patients with hypomobility disorders, respectively. Patients were more likely to receive exercise and manual therapy when initially seeing a non-physician for all diagnostic subgroups (Fig. 3). The exception for manual therapy use was in patients with more than one specific diagnosis, where proportional use by physicians was marginally higher (36.2% vs. 35.4%; Fig. 3). Passive treatments, such as electrical stimulation, ultrasound and heat or cold modalities were the least used of the four non-pharmacological treatment categories, ranging from 11.5% for those with non-specific shoulder pain to 35.9% for those with hypomobility disorders (Figure SA.4). Passive treatments were used most frequently by non-physicians in specialty care settings for all diagnostic subgroups (Supplement Tables SA.1-SA.7). Clinical care setting provided additional context. For example, patients were more likely (2–3 times in some cases) to receive physical therapy and exercise therapy if they were initially seen by a non-physicians in all clinical care settings and for most shoulder diagnostic subgroups (Supplement Tables SA.1-SA.7), but the exceptions were when seeing an emergency physician for rotator cuff, acromioclavicular joint disorders, glenohumeral instability and patients with more than one specific diagnosis. Additionally, patients with more than one specific diagnosis seeing a primary care physician were also marginally more likely to receive exercise and physical therapy (Supplement Figure SA.4; Table SA.7). The highest proportion of patients in all three care settings that eventually received exercise therapy were those in the groups with more than one specific diagnosis, glenohumeral hypomobility, and glenohumeral instability disorders. Overall the proportion receiving exercise ranged from 45.8% (instability in emergency care) to 75.3% (hypomobility in specialty care); Supplement Figure SA.4 and Tables SA.1-SA.7). The use of acupuncture was essentially non-existent regardless of clinician, setting or diagnosis (Tables SA.1-SA.7).

### Diagnostic imaging

**Fig. 4:**
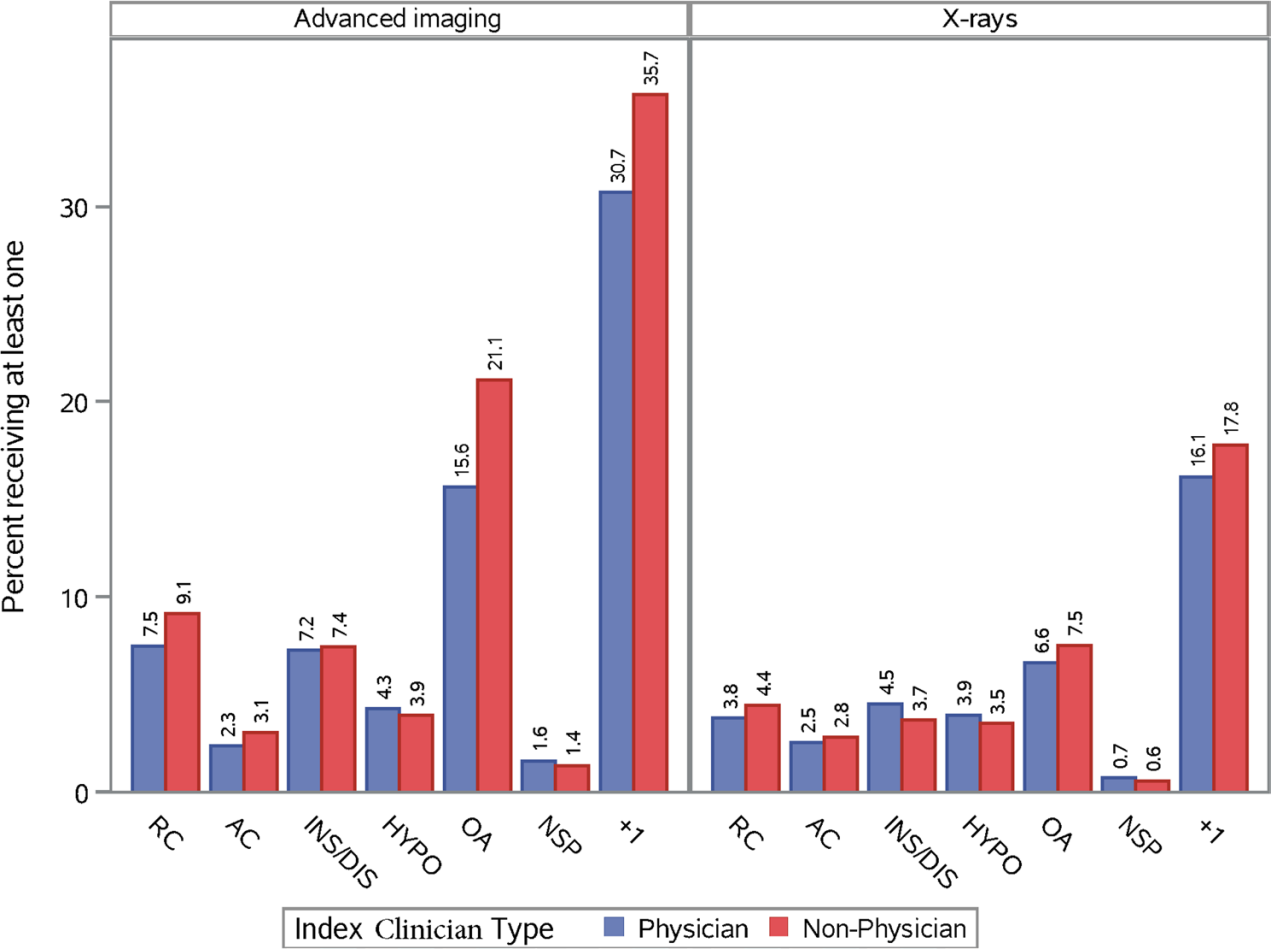
Diagnostic imaging procedures by index clinician type for each shoulder diagnostic category. Note: RC = Rotator Cuff; AC= Acromioclavicular; INS/DIS = instability/dislocation; HYPO = hypomobility (including adhesive capsulitis); OA = Osteoarthritis; NSP = non-specific shoulder pain; +1 = more than 1 specific shoulder diagnostic category

In general, patients with a non-specific diagnosis had the lowest rate of imaging, and patients with more than one diagnosis had the highest rate of imaging; as high as 40.3% (advanced imaging, by non-physicians) among patients initially seen in a primary care setting (Fig. 4 and Supplement Figure SA.5). This trend was similar across care settings regardless of whether a physician or non-physician was seen first. Patients with more than one shoulder diagnosis had higher rates of advanced imaging than radiographs within all settings and regardless of clinician type, except for identical rates of 15.2% (radiograph and advanced imaging) by non-physician clinicians in emergency care settings (Supplemental Table SA.7). For non-specific shoulder diagnosis, radiograph rates were higher than advanced imaging rates if initially seen in emergency care settings but not in primary and specialty care settings(Supplemental Table SA.6). Advanced imaging rates in primary or specialty care settings were equal or higher than radiograph imaging rates for glenohumeral osteoarthritis, hypomobility, instability, and rotator cuff disorders (Supplemental Tables SA.1-SA.5).

Physicians ordered a marginally higher rate of radiographs for hypomobility, instability, and non-specific shoulder disorders, but marginally lower for rotator cuff, acromioclavicular joint disorders, osteoarthritis, and those with more than one specific diagnosis, compared to non-physicians. The patterns were similar for advanced imaging, with wider variability within subgroups (Fig. 4). Within clinical care settings, the proportion of radiograph use was highest by primary care providers for all shoulder disorders except for nonspecific diagnosis (relatively same rates), and glenohumeral instability, which had the highest proportion of radiographic use in emergency care settings (Supplement Figure SA.5). Patients with glenohumeral osteoarthritis and with multiple specific shoulder disorders were more likely to receive at least one advanced imaging procedure when initially seen by a non-physician compared to a physician (21.1% vs. 15.6%, and 35.7% vs. 30.7%, respectively; Fig. 4).

## Discussion

There was variability in shoulder care based on the type of provider the patient initially consulted. However, the specific shoulder disorder and the initial care setting influenced the variability and differences were not equivocal. The majority of care was initiated in primary care settings, which is not suprising considering stepped care is championed in most health systems, with primary care clinicians acting as gatekeepers in order to improve the quality and overall cost of care [28]. Primary care clinicians are charged with initial management, referring to specialists only for complex or unresponsive patients.

Non-physicians initially managed 55.6% of all cases compared to physicians (44.4% of all cases). However, proportional rates varied based on the setting, underscoring the need to consider setting when assessing clinician workload. In emergency care settings, physicians saw more than double the caseload of non-physicians (68.7% versus 31.3%) but in specialty care settings the workload of physicians was approximately a quarter of that of non-physicians (26.4% versus 73.6%). Primary care saw by far the largest proportion of patients. Specifically in this setting, non-physicians are likely to exist in greater numbers than physicians, as they are a relatively less expensive workforce [29], and meant to ‘extend’ the capabilities of physicians [20, 30, 31] during a primary care provider shortage [32, 33]. 

There was other variability based on setting. Physicians in emergency and primary care settings initially saw a greater proportion of patients with rotator cuff disorders, AC joint dysfunction, glenohumeral instability, glenohumeral hypomobility, and patients with more than one diagnosis compared to non-clinicians in the same settings. In contrast, within specialty care settings for patients with the same diagnosis, the proportion of initial care was higher for non-clinicians. The non-specific diagnosis category had marginally different observations. Physicians in emergency care saw a much higher proportion of patients within this subgroup compared to non-physicians (70.9% versus 29.1%). However, initial care by non-physicians was higher in primary care (54.7% versus 45.3%) and specialty care (79.4% versus 20.6%), compared to physicians. This suggests non-physicians are more likely to use a non-specific diagnosis than physicians. Thus, when patients were seen by a physician, they were more likely to get a specific diagnosis, which would have moved them into a specific diagnosis category explaining the higher proportion of patients initially seen by a physician within the other shoulder diagnostic subgroups.

Because of practice pattern variability based on diagnostic subgroup, there was not a straight-forward answer for the hypothesis that patients initially seen in primary care would have lower shoulder-related healthcare utilization rates than patients initially seen in emergency or secondary care settings. For example, patients with only a non-specific diagnosis had the lowest rate of any imaging. Most patients get a non-specific diagnosis first, and then a more specific diagnosis after a work-up that usually results in several additional visits. This is why the patients in the non-specific diagnosis group had the fewest mean number of visits (Fig. 1), and also why they were also less likely to have any imaging studies (Fig. 4). These could have been less severe cases, where the likely scenario is that either the clinician did not deem further workup necessary or the patient did not deem the severity substantial enough to follow-up with any additional visits requested. A recent systematic review synthesized recommendations for 26 clinical practice guidelines for shoulder pain conditions, including imaging [34]. While there was conflicting evidence on the use of initial radiographs, MRI was usually not recommended for early management of shoulder pain. Acetaminophen, NSAIDs, and exercise therapy were all recommended as first-line treatments for all shoulder pain disorders addressed in the guidelines. These recommendations were not adhered to for a large proportion of patients with shoulder pain. This was evidenced by use of only advanced imaging (without prior radiographs) in as high as 25.3% of subgroups (primary care clinicians seeing patients with more than one diagnosis), < 60% of all patients receiving exercise therapy (except for the glenohumeral hypomobility group), and 3–12% of all patients receiving opioids.

The use of non-pharmacological treatment (i.e., therapeutic exercise, physical therapy) was higher for patients initially seen by a non-physician in most shoulder diagnosis groups and within most clinical care settings. This could be explained by the fact that non-physician clinicians included those with less expansive prescription and injection privileges, such as physical and occupational therapists. This could also explain the higher use of passive interventions by this group.

Exercise therapy and evaluation by a physical therapist were the most common non-pharmacological interventions used across all of the shoulder diagnostic subgroups. This is an encouraging finding as this approach is recommended by most clinical practice guidelines and for most shoulder conditions [34]. The highest proportion of exercise therapy occurred when patients saw a non-physician to initiate their shoulder care, likely because physical therapists are non-physicians and exercise is a key component of most physical rehabilitation regimens. The highest proportion of manual therapy utilization came for patients initially seen by primary care providers and for those patients with more than 1 specific shoulder diagnosis. However, for all other diagnostic shoulder subgroups, patients initially seen by non-physician clinicians had the highest proportion of manual therapy use. In primary care, osteopathic physicians are known to utilize manipulative therapy. Non-physician clinicians include physical therapists, who are likely to use manual therapy as it is recommended in several clinical practice guidelines [35, 36], and may improve outcomes when added to an exercise program [37]. 

Corticosteroid injections were the most commonly used pharmacological treatment for all rotator cuff pain-related disorders, glenohumeral OA, hypomobility disorders, AC joint disorders, and individuals within more than one diagnostic category. This treatment was more common when care was initiated with a specialist physician for all categories of shoulder pain, compared to a non-physician. The exception was for individuals with more than one shoulder diagnosis. This is similar to other reports for neck pain, where seeing a specialist as the initial provider significantly increased the odds of injection compared to initially seeing a primary care provider, physical therapist, or chiropractor [9]. Another study found that initiating care for low back pain with a physiatrist also increased the odds of receiving an injection, compared to initiating care with primary care, physical therapist, or chiropractor [38]. Opioids are not typically recommended for initial management of shoulder pain. Opioid prescriptions based on being initially managed by physician versus non-physician clinicians varied based on diagnosis. For rotator cuff, hypermobility, and hypomobility shoulder disorders, initially seeing a physician was more likely to result in an opioid prescription. For acromioclavicular joint disorders, initial management by physicians in emergency care and specialty care, but not primary care, were more likely to result in an opioid prescription than for those initially managed by non-physicians. These results align with those in previously published reports in civilian cohorts. Sun et al. [39] in a cohort of 88,895 opioid-naïve patients with shoulder, neck, knee, or lower back pain, those who saw a non-physician clinician initially for their care (in this case a physical therapist), were 10% less likely to be prescribed opioids.

There are several implications for clinical practice and policy. These results provide a more granular burden of care for the most common types of shoulder disorders, and a direct comparison across shoulder diagnostic subgroups, clinical care settings, and types of clinicians. To date, no studies at the health system level have attempted to characterize and compare medical care based on setting and type of clinician initially managing. Prognosis and practice patterns can vary substantially by diagnosis, and thus care pathways are needed to optimize outcomes for specific diagnostic subgroups. Treatments are often designed to target anatomical and physiological impairments that are specific to a given diagnosis. When epidemiological cohorts are characterized solely by the label ‘shoulder disorder’ or ‘shoulder pain’, or without consideration of clinical care setting, it introduces a great amount of heterogeneity that minimizes potential conclusions and clinical implications.

Future research should focus on assessing the impact of these care patterns on long-term outcomes. For example, to determine whether initial care setting, clinician type, or even geographical location influences long-term use of low-value care, recurrence rates, long-term opioid use, onset of health comorbidities, more intensive pain therapies, or need for surgery. Additionally, three out of every four patients never received a specific diagnostic label, making it difficult to ascertain the appropriateness of the care they received. To make better use of claims and EMR data, improvements in the specificity of diagnosis are necessary. This lack of specificity could have implications for health systems and payers; for example, potential clustering through diagnosis-related groups (DRG) may be challenging. This same phenomenon has been seen with knee disorders [18], where a greater proportion of non-specific diagnoses occurred early on, followed by more specific diagnoses further down the care pathway. The lack of specificity can be challenging to address. In many cases, further workup may be needed before a definitive diagnosis is rendered. Considering the median total number of shoulder visits was only one for patients with this non-specific diagnosis, there were no additional visits on which to confer that final diagnosis. One likely possibility is that many of these were cases presented with lower severity, most often requiring no more than a single visit. These patients had the least amount on average of diagnostic imaging and non-pharmacological treatments. Furthermore, it is not clear if a specific diagnosis will necessarily substantively change the treatment. A more specific diagnostic shoulder label can potentially increase the perceived need for imaging and surgery [40]. 

### Limitations

These results are based on diagnosis codes used in EMR and claims data, and there is likely to be variability across clinicians in their use for various disorders. Diagnostic labels can vary across clinicians, even for the same condition. Therefore, these diagnoses cannot be validated against a more rigorous clinical standard. Additionally, the large majority of cases (almost 75%) received only a non-specific diagnosis within 3 months. Patient-reported outcomes were also not available to help understand the impact of the injuries on pain and function, as well as to help measure injury severity. The MHS has 51 hospitals and 424 clinics, including some international locations. We did not have location data to assess practice variability across geographical locations. These all would greatly improve the relevance of these findings.

## Conclusion

Diagnostic subgroups of shoulder pain and care settings influenced practice variability between physician and non-physician management of shoulder pain. Non-pharmacological treatment was utilized at a marginally higher proportion by non-physician clinicians for all diagnostic shoulder groups, but not always true when considering clinical care setting. The highest proportional use of pharmacological treatments and diagnostic imaging between clinician types also varied greatly based on clinic setting and diagnostic subgroup. However, three out of every four patients had a non-specific shoulder diagnosis. This highlights the need for greater use of specific diagnostic labels within medical record data, to improve the understanding of how shoulder care may differ across diagnostic subgroups. The current system is limited in the extent to which it can asesss care for specific shoulder pathologies.

## Supplementary Information

Supplementary Material 1.

## Data Availability

Data will be made available upon reasonable request and after proper data sharing agreement with the US Defense Health Agency has been obtained. Data sharing agreement applications can be found at health.mil.

## Abbreviations

AC: Acromioclavicular
AHFS: American Hospital Formulary Service
CAPER: Comprehensive Ambulatory Professional Encounter Record
CPT: current procedural terminology
CT: computed tomography
DRG: diagnosis-related groups
EMR: electronic medical record
HYPO: hypomobility
ICD: International Statistical Classification of Diseases and Related Health Problems
MDR: Military Health System Data Repository
MEPRS: Medical Expense and Performance Reporting System
MHS: Miltiary Health System
MRI: magnetic resonance imaging
NSAID: non-steroidal anti-inflammatory drug
NSP: non-specific shoulder pain
OA: osteoarthritis
PDTS: pharmacy data transaction service
PRODLINE: product line
PTSD: post-traumatic stress disorder
RC: rotator cuff
RECORD: REporting of studies Conducted using Observational Routinely-collected Data
STROBE: Strengthening the Reporting of OBservational studies in Epidemiology
SIDR: Standard Inpatient Data Record
TEDI: TRICARE Encounter Data – Institutional
TEDNI: TRICARE Encounter Data – Not Institutional
