# Supplementary material for: Treatment variability for shoulder pain between physician and non-physician clinicians based on initial setting and specific shoulder diagnosis: a health system analysis
Source: BMC Health Serv Res. 2025 Oct 16;25:1370. doi: 10.1186/s12913-025-13175-w (PMC12529843; doi:10.1186/s12913-025-13175-w)
Supplement: Supplementary file 1 — Supplementary Material 1. [file 12913_2025_13175_MOESM1_ESM.pdf]

Supplementary Appendix

TABLE SA1: Healthcare resource utilization for rotator cuff disorders based on provider type and location
TABLE SA2: Healthcare resource utilization for acromioclavicular (AC) joint disorders based on provider type and location
TABLE SA3: Healthcare resource utilization for glenohumeral instability/hypermobility disorders based on provider type and location
TABLE SA4: Healthcare resource utilization for hypomobility/adhesive capsulitis disorders based on provider type and location
TABLE SA5: Healthcare resource utilization for glenohumeral osteoarthritis based on provider type and location
TABLE SA6: Healthcare resource utilization for non-specific shoulder disorders based on provider type and location
TABLE SA7: Healthcare resource utilization for individuals with more than one specific shoulder disorder based on provider type and location

FIGURE SA1: Proportion of case within each diagnostic subgroup across index provider types
FIGURE SA2: Mean number of patient visits for each condition, by provider type.
FIGURE SA3: Proportion of patients receiving pharmacological care by provider type, specific shoulder diagnostic group within setting
FIGURE SA4: Proportion of patients receiving non-pharmacological care by provider type, specific shoulder diagnostic group within setting
FIGURE SA5: Proportion of patients receiving diagnostic imaging by provider type, specific shoulder diagnostic group within Setting

Codes used to identify cohort

TABLE SA.8 ICD Diagnosis Codes – Rotator Cuff Disorders
TABLE SA.9 ICD Diagnosis Codes – Acromioclavicular Joint Dysfunction
TABLE SA.10 ICD Diagnosis Codes - Glenohumeral instability/hypermobility
TABLE SA.11 ICD Diagnosis Codes - Hypomobility/adhesive capsulitis
TABLE SA.12 ICD Diagnosis Codes – Glenohumeral osteoarthritis
TABLE SA.13 ICD Diagnosis Codes – Non-specific shoulder disorders

Table SA1. Healthcare resource utilization for rotator cuff disorders based on provider type and location

Total (N=39,030)	Rotator Cuff Related Disorder					
	Emergency Department		Primary Care		Specialty Care	
	Physician (N=862)	Non-Physician (N=535)	Physician (N=16,787)	Non-Physician (N=14,505)	Physician (N=2575)	Non-Physician (N=3766)
**Shoulder encounters per patient**						
Mean (SD)	3.30 (3.65)	2.69 (2.80)	4.64 (4.56)	4.93 (4.62)	3.94 (4.23)	4.73 (4.41)
Median (Q1, Q3)	2.0 (1.0, 4.0)	2.0 (1.0, 3.0)	3.0 (1.0, 6.0)	3.0 (1.0, 7.0)	2.0 (1.0, 5.0)	3.0 (1.0, 7.0)
Range	1.0-26.0	1.0-20.0	1.0-39.0	1.0-39.0	1.0-36.0	1.0-42.0
**NSAIDS – N (%)**	93 (10.8%)	37 (6.9)	1922 (11.4)	1403 (9.7)	272 (10.6)	356 (9.5)
Mean (SD)	5.34 (3.25)	5.46 (3.66)	5.30 (3.25)	5.25 (3.26)	4.59 (3.04)	4.64 (2.93)
Median (Q1, Q3)	5.0 (3.0, 7.0)	5.0 (3.0, 7.0)	5.0 (3.0, 7.0)	5.0 (3.0, 7.0)	4.0 (2.0, 6.0)	4.0 (2.0, 6.0)
Range	1.0-15.0	1.0-15.0	1.0-25.0	1.0-23.0	1.0-13.0	1.0-17.0
**Analgesics – N (%)**	45 (5.2)	22 (4.1)	993 (5.9)	708 (4.9)	131 (5.1)	183 (4.9)
Mean (SD)	1.76 (0.98)	1.82 (0.91)	1.85 (1.27)	1.87 (1.39)	1.73 (1.25)	1.83 (1.22)
Median (Q1, Q3)	1.0 (1.0, 2.0)	2.0 (1.0, 2.0)	1.0 (1.0, 2.0)	1.0 (1.0, 12.0)	1.0 (1.0, 2.0)	1.0 (1.0, 2.0)
Range	1.0-4.0	1.0-4.0	1.0-9.0	1.0-12.0	1.0-9.0	1.0-7.0
**Muscle relaxers – N (%)**	69 (8.0)	31 (5.8)	1408 (8.4)	1049 (7.2)	176 (6.8)	249 (6.6)
Mean (SD)	2.42 (1.68)	2.45 (1.46)	2.43 (1.77)	2.44 (1.82)	2.31 (1.61)	2.36 (1.84)
Median (Q1, Q3)	2.0 (1.0, 3.0)	2.0 (1.0, 3.0)	2.0 (1.0, 3.0)	2.0 (1.0, 3.0)	2.0 (1.0, 3.0)	2.0 (1.0, 3.0)
Range	1.0-8.0	1.0-7.0	1.0-14.0	1.0-16.0	1.0-8.0	1.0-15.0
**Opioids – N (%)**	72 (8.4)	29 (5.4)	1557 (9.3)	1128 (7.8)	215 (8.3)	290 (7.7)
Mean (SD)	3.65 (2.72)	3.07 (2.12)	3.67 (3.12)	3.65 (3.08)	3.22 (2.59)	3.60 (3.06)
Median (Q1, Q3)	3.0 (1.0, 5.0)	3.0 (2.0, 4.0)	3.0 (1.0, 5.0)	3.0 (1.0, 5.0)	3.0 (1.0, 4.0)	3.0 (1.0, 5.0)
Range	1.0-11.0	1.0-11.0	1.0-33.0	1.0-30.0	1.0-15.0	1.0-23.0
**Benzodiazepine – N (%)**	32 (3.7)	17 (3.2)	672 (4.0)	476 (3.3)	78 (3.0)	115 (3.1)
Mean (SD)	2.47 (2.08)	2.00 (1.50)	2.25 (1.74)	2.24 (1.95)	2.05 (1.38)	2.31 (2.09)
Median (Q1, Q3)	2.0 (1.0, 3.5)	1.0 (1.0, 3.0)	2.0 (1.0, 3.0)	1.0 (1.0, 3.0)	1.0 (1.0, 3.0)	1.0 (1.0, 3.0)
Range	1.0-9.0	1.0-6.0	1.0-12.0	1.0-18.0	1.0-6.0	1.0-12.0
**Opioids and Benzodiazapines – N (%)**	30 (3.5)	14 (2.6)	606 (3.6)	420 (2.9)	66 (2.6)	97 (2.6)
**Steroid joint injections – N (%)**	74 (8.6)	34 (6.4)	2608 (15.5)	2181 (15.0)	706 (27.4)	663 (17.6)
Mean (SD)	1.08 (0.27)	1.09 (0.29)	1.06 (0.25)	1.06 (0.24)	1.07 (0.26)	1.06 (0.25)
Median (Q1, Q3)	1.0 (1.0, 1.0)	1.0 (1.0, 1.0)	1.0 (1.0, 1.0)	1.0 (1.0, 1.0)	1.0 (1.0, 1.0)	1.0 (1.0, 1.0)
Range	1.0-2.0	1.0-2.0	1.0-3.0	1.0-3.0	1.0-3.0	1.0-3.0
**Physical therapy – N (%)**	191 (22.2)	102 (19.1)	7512 (44.7)	6956 (48.0)	761 (29.6)	2618 (69.5)
Mean (SD)	4.20 (3.87)	3.29 (3.01)	4.78 (4.27)	4.69 (4.19)	4.81 (4.46)	4.15 (3.85)

Rotator Cuff Related Disorder						
Total (N=39,030)	Emergency Department		Primary Care		Specialty Care	
	Physician (N=862)	Non-Physician (N=535)	Physician (N=16,787)	Non-Physician (N=14,505)	Physician (N=2575)	Non-Physician (N=3766)
Median (Q1, Q3)	3.0 (1.0, 6.0)	2.0 (1.0, 5.0)	3.0 (1.0, 7.0)	3.0 (1.0, 7.0)	3.0 (1.0, 7.0)	3.0 (1.0, 6.0)
Range	1.0-21.0	1.0-14.0	1.0-33.0	1.0-28.0	1.0-25.0	1.0-28.0
**Passive treatments – N (%)**	91 (10.6)	42 (7.9)	3200 (19.1%)	2937 (20.2%)	517 (20.1)	1071 (28.4)
Mean (SD)	3.82 (3.59)	3.38 (2.63)	4.60 (3.96)	4.50 (3.79)	4.06 (3.81)	3.86 (3.17)
Median (Q1, Q3)	3.0 (1.0, 5.0)	2.0 (2.0, 4.0)	3.0 (2.0, 6.0)	3.0 (2.0, 6.0)	3.0 (1.0, 5.0)	3.0 (1.0, 5.0)
Range	1.0-20.0	1.0-13.0	1.0-32.0	1.0-31.0	1.0-24.0	1.0-25.0
**Exercise Therapy – N (%)**	202 (23.4)	112 (20.9)	7563 (45.1)	6908 (47.6)	965 (37.5)	2417 (64.2)
Mean (SD)	4.37 (3.94)	3.41 (3.04)	4.80 (4.26)	4.76 (4.18)	4.57 (4.22)	4.04 (3.56)
Median (Q1, Q3)	3.0 (1.0, 6.0)	2.0 (1.0, 5.0)	3.0 (1.0, 7.0)	3.0 (1.0, 7.0)	3.0 (1.0, 6.0)	3.0 (1.0, 6.0)
Range	1.0-22.0	1.0-14.0	1.0-33.0	1.0-38.0	1.0-31.0	1.0-25.0
**Manual therapy – N (%)**	93 (10.8)	44 (8.2)	4103 (24.4)	3698 (25.5)	393 (15.3)	1449 (38.5)
Mean (SD)	4.08 (4.21)	2.91 (2.56)	4.31 (4.24)	4.10 (3.96)	3.51 (3.58)	2.85 (2.79)
Median (Q1, Q3)	2.0 (1.0, 5.0)	1.5 (1.0, 5.0)	3.0 (1.0, 6.0)	3.0 (1.0, 6.0)	2.0 (1.0, 5.0)	2.0 (1.0, 4.0)
Range	1.0-21.0	1.0-13.0	1.0-33.0	1.0-25.0	1.0-24.0	1.0-26.0
**Acupuncture – N (%)**	3 (0.3)	3 (0.6)	96 (0.6)	68 (0.5)	40 (1.6)	26 (0.7)
Mean (SD)	1.33 (0.58)	1.00 (0.00)	1.48 (1.11)	1.74 (1.38)	2.23 (2.28)	1.85 (1.76)
Median (Q1, Q3)	1.0 (1.0, 2.0)	1.0 (1.0, 1.0)	1.0 (1.0, 2.0)	1.0 (1.0, 2.0)	1.0 (1.0, 2.0)	1.0 (1.0, 2.0)
Range	1.0-2.0	1.0-1.0	1.0-9.0	1.0-9.0	1.0-11.0	1.0-8.0
**Radiographs – N (%)**	30 (3.5)	11 (2.1)	715 (4.3)	764 (5.3)	29 (1.1)	62 (1.6)
Mean (SD)	1.17 (0.38)	1.00 (0.00)	1.12 (0.35)	1.12 (0.36)	1.03 (0.19)	1.11 (0.32)
Median (Q1, Q3)	1.0 (1.0, 1.0)	1.0 (1.0, 1.0)	1.0 (1.0, 1.0)	1.0 (1.0, 1.0)	1.0 (1.0, 1.0)	1.0 (1.0, 1.0)
Range	1.0-2.0	1.0-1.0	1.0-4.0	1.0-4.0	1.0-2.0	1.0-2.0
**Magnetic Resonance Imaging – N (%)**	35 (4.1)	14 (2.6)	1391 (8.3)	1519 (10.5)	89 (3.5)	176 (4.7)
Mean (SD)	1.17 (0.38)	1.14 (0.36)	1.41 (0.55)	1.42 (0.53)	1.34 (0.50)	1.44 (0.52)
Median (Q1, Q3)	1.0 (1.0, 1.0)	1.0 (1.0, 1.0)	1.0 (1.0, 2.0)	1.0 (1.0, 2.0)	1.0 (1.0, 2.0)	1.0 (1.0, 2.0)
Range	1.0-2.0	1.0-2.0	1.0-4.0	1.0-4.0	1.0-3.0	1.0-3.0
**Arthrogram – N (%)**	1 (0.1)	1 (0.2)	105 (0.6)	105 (0.7)	8 (0.3)	41 (1.1)
Mean (SD)	1.00 (.)	1.00 (.)	1.19 (0.46)	1.27 (0.51)	1.25 (0.46)	1.15 (0.36)
Median (Q1, Q3)	1.0 (1.0, 1.0)	1.0 (1.0, 1.0)	1.0 (1.0, 1.0)	1.0 (1.0, 1.0)	1.0 (1.0, 1.5)	1.0 (1.0, 1.0)
Range	1.0-1.0	1.0-1.0	1.0-4.0	1.0-4.0	1.0-2.0	1.0-2.0
**Computed Tomography scans – N (%)**	0	0	8 (0.0)	9 (0.1)	2 (0.1)	4 (0.1)
Mean (SD)			1.38 (0.52)	1.22 (0.44)	1.50 (0.71)	1.25 (0.50)
Median (Q1, Q3)	.	.	1.0 (1.0, 2.0)	1.0 (1.0, 1.0)	1.5 (1.0, 2.0)	1.0 (1.0, 1.5)
Range			1.0-2.0	1.0-2.0	1.0-2.0	1.0-2.0

Rotator Cuff Related Disorder						
Total (N=39,030)	Emergency Department		Primary Care		Specialty Care	
	Physician (N=862)	Non-Physician (N=535)	Physician (N=16,787)	Non-Physician (N=14,505)	Physician (N=2575)	Non-Physician (N=3766)
**Advanced imaging (MRIs, arthrograms, CT scans) – N (%)**	35 (4.1)	14 (2.6)	1400 (8.3)	1531 (10.6)	92 (3.6)	181 (4.8)
Mean (SD)	1.20 (0.41)	1.21 (0.58)	1.50 (0.71)	1.50 (0.68)	1.43 (0.68)	1.69 (0.83)
Median (Q1, Q3)	1.0 (1.0, 1.0)	1.0 (1.0, 1.0)	1.0 (1.0, 2.0)	1.0 (1.0, 2.0)	1.0 (1.0, 2.0)	1.0 (1.0, 2.0)
Range	1.0-2.0	1.0-3.0	1.0-8.0	1.0-6.0	1.0-4.0	1.0-4.0
**Imaging Use Patterns – N (%)**						
X-ray only	23 (2.7)	9 (1.7)	400 (2.4)	400 (2.8)	14 (0.5)	41 (1.1)
Advanced imaging only	28 (3.2)	12 (2.2)	1085 (6.5)	1167 (8.0)	77 (3.0)	160 (4.2)
X-ray 1st	3 (0.3)	1 (0.2)	144 (0.9)	119 (0.8)	3 (0.1)	5 (0.1)
Advanced imaging 1st	2 (0.2)	1 (0.2)	165 (1.0)	233 (1.6)	2 (0.1)	8 (0.2)
X-ray and Advanced imaging (on index)	2 (0.2)	0 (0.0)	6 (0.0)	12 (0.1)	10 (0.4)	8 (0.2)
No imaging procedures	804 (93.3)	512 (95.7)	14,987 (89.3)	12,574 (86.7)	2469 (95.9)	3544 (94.1)

(report generated on 23JUL2024)
created on 07292024 using program 2_Aim2_20231116.sas

Table SA2. Healthcare resource utilization for acromioclavicular (AC) joint disorders based on provider type and location

	Acromioclavicular Joint Disorder					
Total (N=2237)	Emergency Department		Primary Care		Specialty Care	
	Physician (N=120)	Non-Physician (N=65)	Physician (N=744)	Non-Physician (N=889)	Physician (N=179)	Non-Physician (N=240)
**Shoulder encounters per patient**						
Mean (SD)	3.70 (3.70)	3.20 (3.49)	3.71 (3.83)	4.02 (3.78)	3.26 (3.70)	4.34 (4.26)
Median (Q1, Q3)	2.0 (1.0, 5.0)	2.0 (1.0, 4.0)	2.0 (1.0, 5.0)	3.0 (1.0, 5.0)	2.0 (1.0, 4.0)	3.0 (1.0, 6.0)
Range	1.0-16.0	1.0-19.0	1.0-32.0	1.0-23.0	1.0-24.0	1.0-28.0
**NSAIDS – N (%)**	6 (5.0)	6 (9.2)	39 (5.2)	51 (5.7)	5 (2.8)	6 (2.5)
Mean (SD)	4.50 (1.38)	3.33 (2.07)	5.67 (3.26)	5.41 (3.57)	4.60 (2.30)	3.67 (2.73)
Median (Q1, Q3)	4.0 (4.0, 5.0)	3.5 (1.0, 5.0)	5.0 (3.0, 8.0)	5.0 (3.0, 7.0)	3.0 (3.0, 6.0)	2.5 (2.0, 6.0)
Range	3.0-7.0	1.0-6.0	1.0-14.0	1.0-16.0	3.0-8.0	1.0-8.0
**Analgesics – N (%)**	4 (3.3)	2 (3.1)	21 (2.8)	29 (3.3)	2 (1.1)	2 (0.8)
Mean (SD)	1.75 (1.50)	1.50 (0.71)	2.10 (1.51)	2.24 (2.05)	2.00 (1.41)	1.50 (0.71)
Median (Q1, Q3)	1.0 (1.0, 2.5)	1.5 (1.0, 2.0)	1.0 (1.0, 3.0)	1.0 (1.0, 3.0)	2.0 (1.0, 3.0)	1.5 (1.0, 2.0)
Range	1.0-4.0	1.0-2.0	1.0-6.0	1.0-10.0	1.0-3.0	1.0-2.0
**Muscle relaxers – N (%)**	6 (5.0)	2 (3.1)	28 (3.8)	33 (3.7)	3 (1.7)	5 (2.1)
Mean (SD)	3.33 (1.51)	1.50 (0.71)	2.50 (1.69)	2.36 (1.76)	4.33 (2.08)	2.60 (0.55)
Median (Q1, Q3)	3.0 (2.0, 5.0)	1.5 (1.0, 2.0)	2.0 (1.0, 3.0)	2.0 (1.0, 3.0)	5.0 (2.0, 6.0)	3.0 (2.0, 3.0)
Range	2.0-5.0	1.0-2.0	1.0-7.0	1.0-9.0	2.0-6.0	2.0-3.0
**Opioids – N (%)**	6 (5.0)	3 (4.6)	36 (4.8)	44 (4.9)	6 (3.4)	7 (2.9)
Mean (SD)	4.00 (2.45)	2.67 (0.58)	4.44 (4.46)	3.43 (3.26)	3.83 (2.64)	2.14 (0.90)
Median (Q1, Q3)	4.0 (2.0, 5.0)	3.0 (2.0, 3.0)	3.0 (1.0, 5.0)	2.0 (1.0, 4.0)	3.0 (2.0, 4.0)	2.0 (1.0, 3.0)
Range	1.0-8.0	2.0-3.0	1.0-18.0	1.0-17.0	2.0-9.0	1.0-3.0
**Benzodiazepine – N (%)**	3 (2.5)	1 (1.5)	21 (2.8)	16 (1.8)	4 (2.2)	2 (0.8)
Mean (SD)	3.33 (2.52)	1.00 (.)	1.81 (1.03)	2.81 (2.40)	2.00 (1.41)	3.50 (0.71)
Median (Q1, Q3)	3.0 (1.0, 6.0)	1.0 (1.0, 1.0)	1.0 (1.0, 2.0)	1.5 (1.0, 5.0)	1.5 (1.0, 3.0)	3.5 (3.0, 4.0)
Range	1.0-6.0	1.0-1.0	1.0-4.0	1.0-8.0	1.0-4.0	3.0-4.0
**Opioids and Benzodiazapines – N (%)**	2 (1.7)	1 (1.5)	21 (2.8)	14 (1.6)	4 (2.2)	2 (0.8)
**Steroid joint injections – N (%)**	1 (0.8)	0	55 (7.4)	50 (5.6)	23 (12.8)	14 (5.8)
Mean (SD)	1.0		1.00 (0.00)	1.02 (0.14)	1.00 (0.00)	1.00 (0.00)
Median (Q1, Q3)	1.0 (1.0, 1.0)	.	1.0 (1.0, 1.0)	1.0 (1.0, 1.0)	1.0 (1.0, 1.0)	1.0 (1.0, 1.0)
Range	1.0-1.0		1.0-1.0	1.0-2.0	1.0-1.0	1.0-1.0
**Physical therapy – N (%)**	38 (31.7)	15 (23.1)	292 (39.2)	398 (44.8)	46 (25.7)	157 (65.4)
Mean (SD)	4.18 (3.16)	4.33 (3.48)	3.89 (3.92)	3.80 (3.70)	4.22 (4.08)	3.69 (4.16)

Acromioclavicular Joint Disorder						
Total (N=2237)	Emergency Department		Primary Care		Specialty Care	
	Physician (N=120)	Non-Physician (N=65)	Physician (N=744)	Non-Physician (N=889)	Physician (N=179)	Non-Physician (N=240)
Median (Q1, Q3)	3.0 (2.0, 6.0)	4.0 (1.0, 6.0)	2.0 (1.0, 5.0)	2.0 (1.0, 5.0)	3.0 (1.0, 6.0)	2.0 (1.0, 5.0)
Range	1.0-12.0	1.0-14.0	1.0-24.0	1.0-18.0	1.0-16.0	1.0-28.0
**Passive treatments – N (%)**	15 (12.5)	11 (16.9)	102 (13.7)	148 (16.6)	29 (16.2)	55 (22.9)
Mean (SD)	4.53 (2.85)	3.64 (2.01)	4.19 (4.07)	3.83 (3.76)	2.76 (2.69)	3.96 (3.86)
Median (Q1, Q3)	5.0 (2.0, 8.0)	3.0 (2.0, 5.0)	3.0 (1.0, 5.0)	2.0 (1.0, 5.0)	2.0 (1.0, 3.0)	2.0 (2.0, 5.0)
Range	1.0-9.0	1.0-7.0	1.0-24.0	1.0-20.0	1.0-12.0	1.0-19.0
**Exercise Therapy – N (%)**	37 (30.8)	15 (23.1)	270 (36.3)	364 (40.9)	48 (26.8)	127 (52.9)
Mean (SD)	4.46 (3.46)	4.80 (3.38)	3.86 (3.74)	3.99 (3.75)	4.44 (4.05)	4.19 (4.21)
Median (Q1, Q3)	3.0 (1.0, 7.0)	5.0 (2.0, 6.0)	2.0 (1.0, 5.0)	2.0 (1.0, 5.0)	3.0 (1.5, 6.0)	3.0 (1.0, 6.0)
Range	1.0-12.0	1.0-14.0	1.0-23.0	1.0-22.0	1.0-18.0	1.0-27.0
**Manual therapy – N (%)**	17 (14.2)	5 (7.7)	130 (17.5)	157 (17.7)	14 (7.8)	50 (20.8)
Mean (SD)	3.24 (3.01)	1.20 (0.45)	3.28 (3.18)	3.31 (3.60)	2.71 (3.07)	2.68 (2.49)
Median (Q1, Q3)	2.0 (1.0, 6.0)	1.0 (1.0, 1.0)	2.0 (1.0, 5.0)	2.0 (1.0, 4.0)	1.0 (1.0, 3.0)	1.0 (1.0, 3.0)
Range	1.0-9.0	1.0-2.0	1.0-15.0	1.0-20.0	1.0-11.0	1.0-10.0
**Acupuncture – N (%)**	0	0	3 (0.4)	4 (0.4)	2 (1.1)	0 (0.0)
Mean (SD)			1.00 (0.00)	1.25 (0.50)	1.00 (0.00)	
Median (Q1, Q3)		.	1.0 (1.0, 1.0)	1.0 (1.0, 1.5)	1.0 (1.0, 1.0)	.
Range			1.0-1.0	1.0-2.0	1.0-1.0	
**Radiographs – N (%)**	2 (1.7)	0	24 (3.2)	33 (3.7)	1 (0.6)	1 (0.4)
Mean (SD)	1.00 (0.00)		1.17 (0.48)	1.21 (0.42)	1.00 (.)	1.00 (.)
Median (Q1, Q3)	1.0 (1.0, 1.0)	.	1.0 (1.0, 1.0)	1.0 (1.0, 1.0)	1.0 (1.0, 1.0)	1.0 (1.0, 1.0)
Range	1.0-1.0		1.0-3.0	1.0-2.0	1.0-1.0	1.0-1.0
**Magnetic Resonance Imaging – N (%)**	0	0	23 (3.1)	31 (3.5)	1 (0.6)	5 (2.1)
Mean (SD)			1.39 (0.50)	1.39 (0.50)	1.00 (.)	1.40 (0.55)
Median (Q1, Q3)		.	1.0 (1.0, 2.0)	1.0 (1.0, 2.0)	1.0 (1.0, 1.0)	1.0 (1.0, 1.0)
Range			1.0-2.0	1.0-2.0	1.0-1.0	1.0-2.0
**Arthrogram – N (%)**	0	0	6 (0.8)	2 (0.2)	0	1 (0.4)
Mean (SD)			1.17 (0.41)	1.00 (0.00)		1.00 (.)
Median (Q1, Q3)		.	1.0 (1.0, 1.0)	1.0 (1.0, 1.0)	.	1.0 (1.0, 1.0)
Range			1.0-2.0	1.0-1.0		1.0-1.0
**Computed Tomography scans – N (%)**	0	0	1 (0.1)	0	0	0
Mean (SD)			1.00 (.)			
Median (Q1, Q3)		.	1.0 (1.0, 1.0)	.	.	.
Range			1.0-1.0			

Acromioclavicular Joint Disorder						
Total (N=2237)	Emergency Department		Primary Care		Specialty Care	
	Physician (N=120)	Non-Physician (N=65)	Physician (N=744)	Non-Physician (N=889)	Physician (N=179)	Non-Physician (N=240)
**Advanced imaging (MRIs, arthrograms, CT scans) – N (%)**	0	0	24 (3.2)	32 (3.6)	1 (0.6)	5 (2.1)
Mean (SD)			1.67 (0.96)	1.41 (0.56)	1.00 (.)	1.60 (0.55)
Median (Q1, Q3)		.	1.0 (1.0, 2.5)	1.0 (1.0, 2.0)	1.0 (1.0, 1.0)	2.0 (1.0, 2.0)
Range			1.0-4.0	1.0-3.0	1.0-1.0	1.0-2.0
**Imaging Use Patterns – N (%)**						
X-ray only	2 (1.7)	0	20 (2.7)	26 (2.9)	1 (0.6)	1 (0.4)
Advanced imaging only	0	0	20 (2.7)	25 (2.8)	1 (0.6)	5 (2.1)
X-ray 1st	0	0	2 (0.3)	1 (0.1)	0	0
Advanced imaging 1st	0	0	2 (0.3)	4 (0.4)	0	0
X-ray and Advanced imaging (on index)	0	0	0	2 (0.2)	0	0
No imaging procedures	118 (98.3)	65 (100.0)	700 (94.1)	831 (93.5)	177 (98.9)	234 (97.5)

(report generated on 23JUL2024)
created on 07292024 using program 2_Aim2_20231116.sas

Table SA3. Healthcare resource utilization for glenohumeral instability/dislocation based on provider type and location

	Glenohumeral Instability/Dislocation					
	Emergency Department		Primary Care		Specialty Care	
Total (N=4300)	Physician (N=173)	Non-Physician (N=63)	Physician (N=1487)	Non-Physician (N=1420)	Physician (N=547)	Non-Physician (N=610)
**Shoulder encounters per patient**						
Mean (SD)	5.06 (4.38)	5.25 (4.90)	5.24 (4.69)	5.52 (4.71)	4.85 (4.76)	5.75 (4.88)
Median (Q1, Q3)	4.0 (2.0, 7.0)	4.0 (2.0, 7.0)	4.0 (2.0, 7.0)	4.0 (2.0, 8.0)	3.0 (1.0, 7.0)	4.0 (2.0, 9.0)
Range	1.0-19.0	1.0-24.0	1.0-29.0	1.0-50.0	1.0-31.0	1.0-25.0
**NSAIDS – N (%)**	9 (5.2)	2 (3.2)	96 (6.5)	65 (4.6)	26 (4.8)	22 (3.6)
Mean (SD)	7.44 (4.45)	6.00 (1.41)	4.92 (3.05)	5.48 (2.70)	4.35 (2.54)	4.23 (2.84)
Median (Q1, Q3)	8.0 (4.0, 9.0)	6.0 (5.0, 7.0)	4.0 (3.0, 6.0)	5.0 (4.0, 7.0)	4.0 (2.0, 7.0)	4.0 (2.0, 6.0)
Range	1.0-15.0	5.0-7.0	1.0-15.0	1.0-12.0	1.0-10.0	1.0-11.0
**Analgesics – N (%)**	4 (2.3)	1 (1.6)	44 (3.0)	35 (2.5)	14 (2.6)	12 (2.0)
Mean (SD)	3.00 (1.83)	1.00 (.)	1.57 (1.00)	1.69 (0.90)	1.71 (1.14)	1.58 (1.08)
Median (Q1, Q3)	3.0 (1.5, 4.5)	1.0 (1.0, 1.0)	1.0 (1.0, 2.0)	1.0 (1.0, 2.0)	1.0 (1.0, 2.0)	1.0 (1.0, 2.0)
Range	1.0-5.0	1.0-1.0	1.0-6.0	1.0-4.0	1.0-5.0	1.0-4.0
**Muscle relaxers – N (%)**	7 (4.0)	2 (3.2)	67 (4.5)	51 (3.6)	21 (3.8)	14 (2.3)
Mean (SD)	1.86 (1.21)	1.50 (0.71)	2.37 (1.72)	2.25 (1.61)	2.57 (1.72)	2.36 (1.60)
Median (Q1, Q3)	1.0 (1.0, 3.0)	1.5 (1.0, 2.0)	2.0 (1.0, 3.0)	2.0 (1.0, 3.0)	2.0 (2.0, 3.0)	2.0 (1.0, 3.0)
Range	1.0-4.0	1.0-2.0	1.0-10.0	1.0-7.0	1.0-7.0	1.0-6.0
**Opioids – N (%)**	8 (4.6)	2 (3.2)	75 (5.0)	58 (4.1)	24 (4.4)	17 (2.8)
Mean (SD)	3.00 (1.60)	4.50 (0.71)	4.19 (4.08)	3.00 (2.49)	3.29 (2.84)	3.53 (3.10)
Median (Q1, Q3)	2.5 (2.0, 4.0)	4.5 (4.0, 5.0)	3.0 (1.0, 6.0)	2.0 (1.0, 4.0)	2.0 (1.0, 4.5)	3.0 (2.0, 4.0)
Range	1.0-6.0	4.0-5.0	1.0-20.0	1.0-11.0	1.0-13.0	1.0-14.0
**Benzodiazepine – N (%)**	4 (2.3)	1 (1.6)	31 (2.1)	24 (1.7)	12 (2.2)	6 (1.0)
Mean (SD)	1.50 (0.58)	1.00 (.)	2.19 (1.70)	1.96 (1.68)	2.42 (2.27)	2.00 (1.26)
Median (Q1, Q3)	1.5 (1.0, 2.0)	1.0 (1.0, 1.0)	2.0 (1.0, 3.0)	1.0 (1.0, 2.0)	2.0 (1.0, 2.5)	1.5 (1.0, 3.0)
Range	1.0-2.0	1.0-1.0	1.0-7.0	1.0-8.0	1.0-9.0	1.0-4.0
**Opioids and Benzodiazapines – N (%)**	3 (1.7)	1 (1.6)	30 (2.0)	22 (1.5)	11 (2.0)	4 (0.7)
**Steroid joint injections – N (%)**	5 (2.9)	3 (4.8)	42 (2.8)	36 (2.5)	29 (5.3)	14 (2.3)
Mean (SD)	1.00 (0.00)	1.00 (0.00)	1.02 (0.15)	1.03 (0.17)	1.03 (0.19)	1.14 (0.36)
Median (Q1, Q3)	1.0 (1.0, 1.0)	1.0 (1.0, 1.0)	1.0 (1.0, 1.0)	1.0 (1.0, 1.0)	1.0 (1.0, 1.0)	1.0 (1.0, 1.0)
Range	1.0-1.0	1.0-1.0	1.0-2.0	1.0-2.0	1.0-2.0	1.0-2.0
**Physical therapy – N (%)**	76 (43.9)	25 (39.7)	761 (51.2)	811 (57.1)	215 (39.3)	492 (80.7)
Mean (SD)	4.05 (3.65)	4.52 (4.04)	4.68 (4.02)	4.40 (4.02)	5.05 (4.81)	4.50 (4.17)

Glenohumeral Instability/Dislocation						
Total (N=4300)	Emergency Department		Primary Care		Specialty Care	
	Physician (N=173)	Non-Physician (N=63)	Physician (N=1487)	Non-Physician (N=1420)	Physician (N=547)	Non-Physician (N=610)
Median (Q1, Q3)	2.0 (1.0, 6.0)	3.0 (1.0, 6.0)	3.0 (1.0, 7.0)	3.0 (1.0, 6.0)	3.0 (1.0, 7.0)	3.0 (1.0, 6.0)
Range	1.0-18.0	1.0-14.0	1.0-24.0	1.0-35.0	1.0-26.0	1.0-25.0
**Passive treatments – N (%)**	26 (15.0)	7 (11.1)	326 (21.9)	288 (20.3)	113 (20.7)	173 (28.4)
Mean (SD)	4.00 (2.81)	3.14 (2.79)	4.03 (3.43)	3.57 (2.87)	4.26 (3.72)	3.80 (3.46)
Median (Q1, Q3)	4.0 (1.0, 6.0)	2.0 (1.0, 6.0)	3.0 (1.0, 5.0)	3.0 (1.0, 5.0)	3.0 (2.0, 5.0)	3.0 (1.0, 5.0)
Range	1.0-12.0	1.0-8.0	1.0-22.0	1.0-16.0	1.0-23.0	1.0-19.0
**Exercise Therapy – N (%)**	80 (46.2)	28 (44.4)	739 (49.7)	791 (55.7)	224 (41.0)	443 (72.6)
Mean (SD)	4.18 (3.46)	4.75 (4.17)	4.87 (4.00)	4.73 (4.00)	4.98 (4.39)	4.48 (3.95)
Median (Q1, Q3)	3.0 (1.0, 6.5)	4.0 (1.0, 6.0)	4.0 (2.0, 7.0)	4.0 (2.0, 7.0)	4.0 (2.0, 7.0)	3.0 (1.0, 6.0)
Range	1.0-18.0	1.0-16.0	1.0-24.0	1.0-39.0	1.0-25.0	1.0-25.0
**Manual therapy – N (%)**	22 (12.7)	8 (12.7)	255 (17.1)	255 (18.0)	87 (15.9)	164 (26.9)
Mean (SD)	2.95 (2.30)	3.13 (3.36)	3.59 (3.37)	3.25 (3.34)	2.87 (2.78)	2.29 (2.25)
Median (Q1, Q3)	2.0 (1.0, 4.0)	1.5 (1.0, 5.0)	2.0 (1.0, 5.0)	2.0 (1.0, 4.0)	2.0 (1.0, 4.0)	1.0 (1.0, 3.0)
Range	1.0-8.0	1.0-9.0	1.0-17.0	1.0-23.0	1.0-15.0	1.0-20.0
**Acupuncture – N (%)**	0	0	5 (0.3)	3 (0.2)	3 (0.5)	1 (0.2)
Mean (SD)			1.40 (0.55)	1.33 (0.58)	1.00 (0.00)	1.00 (.)
Median (Q1, Q3)			1.0 (1.0, 2.0)	1.0 (1.0, 2.0)	1.0 (1.0, 1.0)	1.0 (1.0, 1.0)
Range			1.0-2.0	1.0-2.0	1.0-1.0	1.0-1.0
**Radiographs – N (%)**	10 (5.8)	5 (7.9)	74 (5.0)	62 (4.4)	18 (3.3)	11 (1.8)
Mean (SD)	1.00 (0.00)	1.20 (0.45)	1.09 (0.29)	1.16 (0.37)	1.28 (0.57)	1.09 (0.30)
Median (Q1, Q3)	1.0 (1.0, 1.0)	1.0 (1.0, 1.0)	1.0 (1.0, 1.0)	1.0 (1.0, 1.0)	1.0 (1.0, 1.0)	1.0 (1.0, 1.0)
Range	1.0-1.0	1.0-2.0	1.0-2.0	1.0-2.0	1.0-3.0	1.0-2.0
**Magnetic Resonance Imaging – N (%)**	8 (4.6)	1 (1.6)	131 (8.8)	118 (8.3)	18 (3.3)	34 (5.6)
Mean (SD)	1.25 (0.46)	1.00 (.)	1.46 (0.57)	1.47 (0.52)	1.22 (0.43)	1.35 (0.65)
Median (Q1, Q3)	1.0 (1.0, 1.5)	1.0 (1.0, 1.0)	1.0 (1.0, 2.0)	1.0 (1.0, 2.0)	1.0 (1.0, 1.0)	1.0 (1.0, 2.0)
Range	1.0-2.0	1.0-1.0	1.0-4.0	1.0-3.0	1.0-2.0	1.0-4.0
**Arthrogram – N (%)**	1 (0.6)	0	26 (1.7)	28 (2.0)	4 (0.7)	7 (1.1)
Mean (SD)	2.00 (.)		1.23 (0.51)	1.21 (0.50)	1.00 (0.00)	1.00 (0.00)
Median (Q1, Q3)	2.0 (2.0, 2.0)	.	1.0 (1.0, 1.0)	1.0 (1.0, 1.0)	1.0 (1.0, 1.0)	1.0 (1.0, 1.0)
Range	2.0-2.0		1.0-3.0	1.0-3.0	1.0-1.0	1.0-1.0
**Computed Tomography scans – N (%)**	0	1 (1.6)	5 (0.3)	1 (0.1)	0	2 (0.3)
Mean (SD)		2.00 (.)	1.20 (0.45)	1.00 (.)		1.00 (0.00)
Median (Q1, Q3)	.	2.0 (2.0-2.0)	1.0 (1.0, 1.0)	1.0 (1.0, 1.0)	.	1.0 (1.0, 1.0)
Range		2.0-2.0	1.0-2.0	1.0-1.0		1.0-1.0

Glenohumeral Instability/Dislocation						
	Emergency Department		Primary Care		Specialty Care	
Total (N=4300)	Physician (N=173)	Non-Physician (N=63)	Physician (N=1487)	Non-Physician (N=1420)	Physician (N=547)	Non-Physician (N=610)
**Advanced imaging**						
**(MRIs, arthrograms, CT scans) – N (%)**	8 (4.6)	2 (3.2)	138 (9.3)	120 (8.5)	18 (3.3)	35 (5.7)
Mean (SD)	1.50 (1.07)	1.50 (0.71)	1.66 (0.90)	1.74 (0.87)	1.44 (0.70)	1.57 (0.85)
Median (Q1, Q3)	1.0 (1.0, 1.5)	1.5 (1.0, 2.0)	1.0 (1.0, 2.0)	2.0 (1.0, 2.0)	1.0 (1.0, 2.0)	1.0 (1.0, 2.0)
Range	1.0-4.0	1.0-2.0	1.0-7.0	1.0-5.0	1.0-3.0	1.0-4.0
**Imaging Use Patterns – N (%)**						
X-ray only	8 (4.6)	3 (4.8)	41 (2.8)	38 (2.7)	11 (2.0)	4 (0.7)
Advanced imaging only	6 (3.5)	0	105 (7.1)	96 (6.8)	11 (2.0)	28 (4.6)
X-ray 1st	2 (1.2)	1 (1.6)	18 (1.2)	12 (0.8)	1 (0.2)	2 (0.3)
Advanced imaging 1st	0	0	14 (0.9)	10 (0.7)	0	1 (0.2)
X-ray and Advanced imaging (on index)	0	1 (1.6)	1 (0.1)	2 (0.1)	6 (1.1)	4 (0.7)
No imaging procedures	157 (90.8)	58 (92.1)	1308 (88.0)	1262 (88.9)	518 (94.7)	571 (93.6)

(report generated on 23JUL2024)
created on 07292024 using program 2_Aim2_20231116.sas

Table SA4. Healthcare resource utilization for hypomobility/adhesive capsulitis based on provider type and location

Total (N=4528)	Hypomobility/Adhesive Capsulitis					
	Emergency Department		Primary Care		Specialty Care	
	Physician (N=71)	Non-Physician (N=31)	Physician (N=1862)	Non-Physician (N=1505)	Physician (N=184)	Non-Physician (N=875)
**Shoulder encounters per patient**						
Mean (SD)	5.13 (4.65)	4.87 (3.67)	6.77 (5.66)	7.45 (5.58)	5.22 (5.56)	6.17 (6.38)
Median (Q1, Q3)	3.0 (2.0, 7.0)	5.0 (2.0, 6.0)	5.0 (2.0, 10.0)	6.0 (2.0, 11.0)	3.0 (1.0, 7.5)	4.0 (1.0, 9.0)
Range	1.0-25.0	1.0-17.0	1.0-35.0	1.0-31.0	1.0-32.0	1.0-43.0
**NSAIDS – N (%)**	9 (12.7)	1 (3.2)	281 (15.1)	183 (12.2)	27 (14.7)	95 (10.9)
Mean (SD)	5.56 (2.96)	5.00 (.)	5.69 (3.50)	5.31 (3.27)	5.22 (3.27)	4.65 (3.22)
Median (Q1, Q3)	6.0 (5.0, 6.0)	5.0 (5.0, 5.0)	5.0 (3.0, 7.0)	5.0 (3.0, 7.0)	5.0 (2.0, 7.0)	4.0 (2.0, 7.0)
Range	1.0-11.0	5.0-5.0	1.0-20.0	1.0-20.0	1.0-13.0	1.0-17.0
**Analgesics – N (%)**	5 (7.0)	1 (3.2)	140 (7.5)	99 (6.6)	13 (7.1)	45 (5.1)
Mean (SD)	1.80 (1.30)	2.00 (.)	1.91 (1.31)	2.02 (1.25)	2.54 (1.39)	1.89 (1.03)
Median (Q1, Q3)	1.0 (1.0, 2.0)	2.0 (2.0, 2.0)	1.0 (1.0, 2.0)	2.0 (1.0, 3.0)	2.0 (1.0, 4.0)	2.0 (1.0, 3.0)
Range	1.0-4.0	2.0-2.0	1.0-7.0	1.0-6.0	1.0-5.0	1.0-5.0
**Muscle relaxers – N (%)**	6 (8.5)	1 (3.2)	217 (11.7)	133 (8.8)	22 (12.0)	65 (7.4)
Mean (SD)	4.00 (2.37)	1.00 (.)	2.49 (1.72)	2.32 (1.78)	2.41 (2.15)	2.11 (1.29)
Median (Q1, Q3)	4.0 (2.0, 6.0)	1.0 (1.0, 1.0)	2.0 (1.0, 3.0)	2.0 (1.0, 3.0)	2.0 (1.0, 3.0)	2.0 (1.0, 3.0)
Range	1.0-7.0	1.0-1.0	1.0-10.0	1.0-11.0	1.0-10.0	1.0-6.0
**Opioids – N (%)**	8 (11.3)	1 (3.2)	233 (12.5)	143 (9.5)	27 (14.7)	84 (9.6)
Mean (SD)	3.00 (2.39)	1.00 (.)	3.82 (3.24)	3.57 (2.96)	3.41 (3.43)	2.92 (2.08)
Median (Q1, Q3)	2.0 (1.0, 5.0)	1.0 (1.0, 1.0)	3.0 (1.0, 5.0)	3.0 (1.0, 5.0)	2.0 (1.0, 4.0)	2.5 (1.0, 4.0)
Range	1.0-7.0	1.0-1.0	1.0-18.0	1.0-16.0	1.0-14.0	1.0-10.0
**Benzodiazepine – N (%)**	4 (5.6)	1 (3.2)	89 (4.8)	56 (3.7)	10 (5.4)	30 (3.4)
Mean (SD)	1.50 (1.00)	1.00 (.)	2.43 (2.92)	2.23 (1.78)	1.70 (1.49)	2.70 (2.12)
Median (Q1, Q3)	1.0 (1.0, 2.0)	1.0 (1.0, 1.0)	2.0 (1.0, 3.0)	1.0 (1.0, 3.0)	1.0 (1.0, 1.0)	2.0 (1.0, 3.0)
Range	1.0-3.0	1.0-1.0	1.0-25.0	1.0-10.0	1.0-5.0	1.0-11.0
**Opioids and Benzodiazapines – N (%)**	4 (5.6)	1 (3.2)	81 (4.4)	46 (3.1)	10 (5.4)	29 (3.3)
**Steroid joint injections – N (%)**	7 (9.9)	6 (19.4)	298 (16.0)	203 (13.5)	40 (21.7)	58 (6.6)
Mean (SD)	1.00 (0.00)	1.00 (0.00)	1.06 (0.25)	1.07 (0.26)	1.05 (0.22)	1.10 (0.31)
Median (Q1, Q3)	1.0 (1.0, 1.0)	1.0 (1.0, 1.0)	1.0 (1.0, 1.0)	1.0 (1.0, 1.0)	1.0 (1.0, 1.0)	1.0 (1.0, 1.0)
Range	1.0-1.0	1.0-1.0	1.0-3.0	1.0-2.0	1.0-2.0	1.0-2.0
**Physical therapy – N (%)**	27 (38.0)	15 (48.4)	1164 (62.5)	968 (64.3)	77 (41.8)	661 (75.5)
Mean (SD)	4.19 (3.62)	3.73 (3.43)	5.88 (5.19)	6.13 (4.97)	5.81 (5.22)	5.40 (5.68)

Hypomobility/Adhesive Capsulitis						
Total (N=4528)	Emergency Department		Primary Care		Specialty Care	
	Physician (N=71)	Non-Physician (N=31)	Physician (N=1862)	Non-Physician (N=1505)	Physician (N=184)	Non-Physician (N=875)
Median (Q1, Q3)	3.0 (1.0, 7.0)	3.0 (1.0, 4.0)	4.0 (2.0, 9.0)	5.0 (2.0, 9.0)	4.0 (1.0, 9.0)	3.0 (1.0, 8.0)
Range	1.0-13.0	1.0-13.0	1.0-31.0	1.0-24.0	1.0-28.0	1.0-41.0
**Passive treatments – N (%)**	19 (26.8)	4 (12.9)	637 (34.2)	586 (38.9)	37 (20.1)	343 (39.2)
Mean (SD)	4.68 (3.86)	4.50 (3.11)	5.51 (4.46)	5.54 (4.29)	4.54 (3.67)	4.80 (4.46)
Median (Q1, Q3)	3.0 (1.0, 7.0)	4.5 (2.0, 7.0)	4.0 (2.0, 8.0)	4.0 (2.0, 8.0)	3.0 (2.0, 8.0)	3.0 (2.0, 6.0)
Range	1.0-13.0	1.0-8.0	1.0-29.0	1.0-23.0	1.0-14.0	1.0-30.0
**Exercise Therapy – N (%)**	32 (45.1)	15 (48.4)	1281 (68.8)	1096 (72.8)	87 (47.3)	710 (81.1)
Mean (SD)	5.34 (4.53)	4.13 (3.54)	6.14 (5.02)	6.53 (4.87)	5.75 (4.99)	5.44 (5.58)
Median (Q1, Q3)	3.5 (1.5, 8.5)	3.0 (1.0, 6.0)	5.0 (2.0, 9.0)	6.0 (2.0, 10.0)	5.0 (2.0, 8.0)	3.0 (1.0, 8.0)
Range	1.0-19.0	1.0-13.0	1.0-31.0	1.0-28.0	1.0-25.0	1.0-41.0
**Manual therapy – N (%)**	22 (31.0)	8 (25.8)	922 (49.5)	796 (52.9)	59 (32.1)	525 (60.0)
Mean (SD)	4.73 (3.78)	4.25 (4.03)	5.71 (4.77)	5.75 (4.65)	5.15 (4.89)	4.55 (4.91)
Median (Q1, Q3)	4.0 (1.0, 6.0)	3.0 (1.5, 5.5)	4.0 (2.0, 8.0)	4.0 (2.0, 8.0)	3.0 (2.0, 7.0)	3.0 (1.0, 6.0)
Range	1.0-12.0	1.0-13.0	1.0-30.0	1.0-25.0	1.0-24.0	1.0-40.0
**Acupuncture – N (%)**	0	1 (3.2)	18 (1.0)	12 (0.8)	11 (6.0)	6 (0.7)
Mean (SD)		1.00 (.)	2.06 (1.30)	2.08 (2.35)	1.45 (0.82)	1.50 (0.84)
Median (Q1, Q3)	.	1.0 (1.0, 1.0)	1.5 (1.0, 3.0)	1.0 (1.0, 2.0)	1.0 (1.0, 2.0)	1.0 (1.0, 2.0)
Range		1.0-1.0	1.0-5.0	1.0-9.0	1.0-3.0	1.0-3.0
**Radiographs – N (%)**	3 (4.2)	1 (3.2)	80 (4.3)	78 (5.2)	1 (0.5)	6 (0.7)
Mean (SD)	1.67 (0.58)	1.00 (.)	1.09 (0.28)	1.09 (0.29)	1.00 (.)	1.00 (0.00)
Median (Q1, Q3)	2.0 (1.0, 2.0)	1.0 (1.0, 1.0)	1.0 (1.0, 1.0)	1.0 (1.0, 1.0)	1.0 (1.0, 1.0)	1.0 (1.0, 1.0)
Range	1.0-2.0	1.0-1.0	1.0-2.0	1.0-2.0	1.0-1.0	1.0-1.0
**Magnetic Resonance Imaging – N (%)**	1 (1.4)	0	86 (4.6)	86 (5.7)	0	9 (1.0)
Mean (SD)	1.00 (.)		1.42 (0.52)	1.43 (0.50)		1.33 (0.50)
Median (Q1, Q3)	1.0 (1.0-1.0)	.	1.0 (1.0-2.0)	1.0 (1.0-2.0)	.	1.0 (1.0-2.0)
Range	1.0-1.0		1.0-3.0	1.0-2.0		1.0-2.0
**Arthrogram – N (%)**	0	0	10 (0.5)	10 (0.7)	0	0
Mean (SD)			1.10 (0.32)	1.20 (0.42)		
Median (Q1, Q3)	.	.	1.0 (1.0, 1.0)	1.0 (1.0, 1.0)	.	.
Range			1.0-2.0	1.0-2.0		
**Computed Tomography scans – N (%)**	0	0	2 (0.1)	0	0	0
Mean (SD)			1.50 (0.71)			
Median (Q1, Q3)	.	.	1.5 (1.0, 2.0)	.	.	.
Range			1.0-2.0			

Hypomobility/Adhesive Capsulitis						
Total (N=4528)	Emergency Department		Primary Care		Specialty Care	
	Physician (N=71)	Non-Physician (N=31)	Physician (N=1862)	Non-Physician (N=1505)	Physician (N=184)	Non-Physician (N=875)
**Advanced imaging (MRIs, arthrograms, CT scans) – N (%)**	1 (1.4)	0 (0.0)	90 (4.8)	86 (5.7)	0 (0.0)	9 (1.0)
Mean (SD)	1.00 (.)		1.51 (0.69)	1.57 (0.70)		1.33 (0.50)
Median (Q1, Q3)	1.0 (1.0, 1.0)	.	1.0 (1.0, 2.0)	1.0 (1.0, 2.0)	.	1.0 (1.0, 2.0)
Range	1.0-1.0		1.0-4.0	1.0-4.0		1.0-2.0
**Imaging Use Patterns – N (%)**						
X-ray only	3 (4.2)	1 (3.2)	70 (3.8)	57 (3.8)	1 (0.5)	6 (0.7)
Advanced imaging only	1 (1.4)	0	80 (4.3)	65 (4.3)	0	9 (1.0)
X-ray 1st	0	0	2 (0.1)	7 (0.5)	0	0
Advanced imaging 1st	0	0	5 (0.3)	14 (0.9)	0	0
X-ray and Advanced imaging (on index)	0	0	3 (0.2)	0 (0.0)	0	0
No imaging procedures	67 (94.4)	30 (96.8)	1702 (91.4)	1362 (90.5)	183 (99.5)	860 (98.3)

(report generated on 23JUL2024)
created on 07292024 using program 2_Aim2_20231116.sas

Table SA5. Healthcare resource utilization for glenohumeral osteoarthritis based on provider type and location

Total (N=4910)	Glenohumeral Osteoarthritis					
	Emergency Department		Primary Care		Specialty Care	
	Physician (N=56)	Non-Physician (N=49)	Physician (N=2028)	Non-Physician (N=2028)	Physician (N=397)	Non-Physician (N=352)
**Shoulder encounters per patient**						
Mean (SD)	3.39 (3.30)	3.55 (3.03)	4.47 (3.89)	4.90 (3.99)	3.21 (3.45)	4.78 (4.32)
Median (Q1, Q3)	2.0 (1.0, 4.0)	2.0 (1.0, 5.0)	3.0 (2.0, 5.0)	4.0 (2.0, 6.0)	2.0 (1.0, 3.0)	3.0 (2.0, 6.0)
Range	1.0-16.0	1.0-11.0	1.0-29.0	1.0-29.0	1.0-22.0	1.0-23.0
**NSAIDS – N (%)**	7 (12.5)	11 (22.4)	285 (14.1)	305 (15.0)	37 (9.3)	47 (13.4)
Mean (SD)	6.43 (5.41)	4.64 (2.87)	4.96 (2.98)	5.02 (3.28)	4.41 (3.71)	5.38 (4.41)
Median (Q1, Q3)	6.0 (1.0, 12.0)	3.0 (3.0, 6.0)	5.0 (3.0, 7.0)	4.0 (3.0, 6.0)	3.0 (1.0, 7.0)	5.0 (1.0, 7.0)
Range	1.0-15.0	1.0-10.0	1.0-17.0	1.0-18.0	1.0-18.0	1.0-22.0
**Analgesics – N (%)**	3 (5.4)	6 (12.2)	145 (7.1)	159 (7.8)	14 (3.5)	19 (5.4)
Mean (SD)	2.67 (2.08)	1.67 (1.21)	1.97 (1.47)	1.78 (1.15)	1.50 (0.65)	1.53 (0.61)
Median (Q1, Q3)	2.0 (1.0-5.0)	1.0 (1.0-2.0)	2.0 (1.0-2.0)	1.0 (1.0-2.0)	1.0 (1.0-2.0)	1.0 (1.0-2.0)
Range	1.0-5.0	1.0-4.0	1.0-10.0	1.0-5.0	1.0-3.0	1.0-3.0
**Muscle relaxers – N (%)**	5 (8.9)	7 (14.3)	216 (10.7)	227 (11.2)	26 (6.5)	34 (9.7)
Mean (SD)	2.00 (1.22)	2.86 (1.21)	2.32 (1.86)	2.53 (2.14)	2.35 (1.74)	2.53 (1.96)
Median (Q1, Q3)	2.0 (1.0, 2.0)	2.0 (2.0, 4.0)	2.0 (1.0, 3.0)	2.0 (1.0, 3.0)	1.0 (1.0, 4.0)	2.0 (1.0, 3.0)
Range	1.0-4.0	2.0-5.0	1.0-13.0	1.0-18.0	1.0-6.0	1.0-11.0
**Opioids – N (%)**	6 (10.7)	11 (22.4)	245 (12.1)	248 (12.2)	31 (7.8)	41 (11.6)
Mean (SD)	2.67 (1.75)	1.91 (1.22)	3.71 (3.15)	3.45 (2.99)	3.68 (3.17)	3.05 (2.80)
Median (Q1, Q3)	2.0 (2.0, 3.0)	2.0 (1.0, 2.0)	3.0 (1.0, 5.0)	3.0 (1.0, 5.0)	3.0 (1.0, 5.0)	2.0 (1.0, 4.0)
Range	1.0-6.0	1.0-5.0	1.0-16.0	1.0-19.0	1.0-12.0	1.0-15.0
**Benzodiazepine – N (%)**	1 (1.8)	2 (4.1)	94 (4.6)	100 (4.9)	12 (3.0)	15 (4.3)
Mean (SD)	3.00 (.)	1.50 (0.71)	2.37 (1.85)	2.14 (1.87)	2.75 (1.60)	2.53 (2.10)
Median (Q1, Q3)	3.0 (3.0, 3.0)	1.5 (1.0, 2.0)	2.0 (1.0, 3.0)	1.0 (1.0, 3.0)	2.5 (1.5, 3.5)	2.0 (1.0, 4.0)
Range	3.0-3.0	1.0-2.0	1.0-9.0	1.0-13.0	1.0-6.0	1.0-7.0
**Opioids and Benzodiazapines – N (%)**	1 (1.8)	2 (4.1)	84 (4.1)	90 (4.4)	11 (2.8)	12 (3.4)
**Steroid joint injections – N (%)**	10 (17.9)	10 (20.4)	404 (19.9)	406 (20.0)	155 (39.0)	130 (36.9)
Mean (SD)	1.10 (0.32)	1.00 (0.00)	1.09 (0.36)	1.08 (0.28)	1.10 (0.31)	1.16 (0.55)
Median (Q1, Q3)	1.0 (1.0, 1.0)	1.0 (1.0, 1.0)	1.0 (1.0, 1.0)	1.0 (1.0, 1.0)	1.0 (1.0, 1.0)	1.0
Range	1.0-2.0	1.0-1.0	1.0-6.0	1.0-2.0	1.0-2.0	1.0-6.0
**Physical therapy – N (%)**	11 (19.6)	12 (24.5)	693 (34.2)	757 (37.3)	78 (19.6)	187 (53.1)
Mean (SD)	3.82 (4.17)	2.83 (2.55)	4.05 (3.93)	4.48 (3.86)	5.29 (4.88)	4.13 (3.79)

Total (N=4910)	Glenohumeral Osteoarthritis					
	Emergency Department		Primary Care		Specialty Care	
	Physician (N=56)	Non-Physician (N=49)	Physician (N=2028)	Non-Physician (N=2028)	Physician (N=397)	Non-Physician (N=352)
Median (Q1, Q3)	1.0 (1.0, 8.0)	1.5 (1.0, 4.5)	2.0 (1.0, 6.0)	3.0 (1.0, 7.0)	3.5 (1.0, 8.0)	2.0 (1.0, 6.0)
Range	1.0-12.0	1.0-8.0	1.0-25.0	1.0-22.0	1.0-21.0	1.0-16.0
**Passive treatments – N (%)**	4 (7.1)	8 (16.3)	258 (12.7)	292 (14.4)	45 (11.3)	73 (20.7)
Mean (SD)	4.50 (2.89)	3.25 (1.58)	4.44 (3.81)	4.32 (3.60)	4.00 (3.24)	3.68 (2.90)
Median (Q1, Q3)	4.5 (2.5, 6.5)	3.5 (2.0, 4.0)	3.0 (2.0, 6.0)	3.0 (2.0, 6.0)	4.0 (1.0, 6.0)	3.0 (1.0, 5.0)
Range	1.0-8.0	1.0-6.0	1.0-24.0	1.0-26.0	1.0-17.0	1.0-12.0
**Exercise Therapy – N (%)**	12 (21.4)	15 (30.6)	657 (32.4)	734 (36.2)	84 (21.2)	151 (42.9)
Mean (SD)	4.33 (3.87)	3.20 (2.21)	4.19 (3.81)	4.52 (3.87)	4.98 (4.43)	4.38 (3.74)
Median (Q1, Q3)	3.0 (1.0, 6.5)	3.0 (1.0, 4.0)	3.0 (1.0, 6.0)	3.0 (1.0, 7.0)	4.0 (1.0, 7.0)	3.0 (1.0, 7.0)
Range	1.0-12.0	1.0-8.0	1.0-25.0	1.0-27.0	1.0-21.0	1.0-21.0
**Manual therapy – N (%)**	6 (10.7)	8 (16.3)	345 (17.0)	401 (19.8)	35 (8.8)	92 (26.1)
Mean (SD)	2.50 (2.35)	2.13 (1.36)	3.64 (3.56)	3.80 (3.41)	3.57 (3.87)	3.28 (3.34)
Median (Q1, Q3)	1.5 (1.0, 3.0)	2.0 (1.0, 2.5)	2.0 (1.0, 5.0)	2.0 1.0, 5.0)	2.0 1.0, 5.0)	2.0 1.0, 5.0)
Range	1.0-7.0	1.0-5.0	1.0-24.0	1.0-20.0	1.0-17.0	1.0-19.0
**Acupuncture – N (%)**	0	2 (4.1)	13 (0.6)	7 (0.3)	1 (0.3)	4 (1.1)
Mean (SD)		2.00 (1.41)	1.23 (0.60)	1.57 (0.79)	1.00 (.)	3.25 (2.63)
Median (Q1, Q3)	.	2.0 (1.0, 3.0)	1.0 (1.0, 1.0)	1.0 (1.0, 2.0)	1.0 (1.0, 1.0)	2.5 (1.5, 5.0)
Range		1.0-3.0	1.0-3.0	1.0-3.0	1.0-1.0	1.0-7.0
**Radiographs – N (%)**	1 (1.8)	2 (4.1)	158 (7.8)	172 (8.5)	7 (1.8)	9 (2.6)
Mean (SD)	1.00 (.)	1.00 (0.00)	1.24 (0.46)	1.15 (0.36)	1.00 (0.00)	1.44 (0.53)
Median (Q1, Q3)	1.0 (1.0, 1.0)	1.0 (1.0, 1.0)	1.0 (1.0, 1.0)	1.0 (1.0, 1.0)	1.0 (1.0, 1.0)	1.0 (1.0, 2.0)
Range	1.0-1.0	1.0-1.0	1.0-3.0	1.0-2.0	1.0-1.0	1.0-2.0
**Magnetic Resonance Imaging – N (%)**	2 (3.6)	1 (2.0)	367 (18.1)	477 (23.5)	18 (4.5)	31 (8.8)
Mean (SD)	1.50 (0.71)	1.00 (.)	1.47 (0.56)	1.43 (0.53)	1.39 (0.50)	1.48 (0.68)
Median (Q1, Q3)	1.5 (1.0, 2.0)	1.0 (1.0, 1.0)	1.0 (1.0, 2.0)	1.0 (1.0, 2.0)	1.0 (1.0, 2.0)	1.0 (1.0, 2.0)
Range	1.0-2.0	1.0-1.0	1.0-4.0	1.0-4.0	1.0-2.0	1.0-4.0
**Arthrogram – N (%)**	1 (1.8)	0	29 (1.4)	22 (1.1)	3 (0.8)	6 (1.7)
Mean (SD)	1.00 (.)		1.21 (0.49)	1.32 (0.57)	1.00 (0.00)	1.50 (0.84)
Median (Q1, Q3)	1.0 (1.0, 1.0)	.	1.0 (1.0, 1.0)	1.0 (1.0, 2.0)	1.0 (1.0, 1.0)	1.0 (1.0, 2.0)
Range	1.0-1.0		1.0-3.0	1.0-3.0	1.0-1.0	1.0-3.0
**Computed Tomography scans – N (%)**	0	0	5 (0.2)	8 (0.4)	0	0
Mean (SD)			1.60 (0.55)	1.00 (0.00)		
Median (Q1, Q3)			2.0 (1.0, 2.0)	1.0 (1.0, 1.0)	.	.
Range			1.0-2.0	1.0-1.0		

Glenohumeral Osteoarthritis						
Total (N=4910)	Emergency Department		Primary Care		Specialty Care	
	Physician (N=56)	Non-Physician (N=49)	Physician (N=2028)	Non-Physician (N=2028)	Physician (N=397)	Non-Physician (N=352)
**Advanced imaging (MRIs, arthrograms, CT scans) – N (%)**	3 (5.4)	1 (2.0)	370 (18.2)	483 (23.8)	18 (4.5)	31 (8.8)
Mean (SD)	1.33 (0.58)	1.00 (.)	1.58 (0.75)	1.49 (0.67)	1.56 (0.62)	1.77 (1.28)
Median (Q1, Q3)	1.0 (1.0-2.0)	1.0 (1.0-1.0)	1.0 (1.0-6.0)	1.0 (1.0-6.0)	1.5 (1.0-3.0)	1.0 (1.0-2.0)
Range	1.0-2.0	1.0-1.0	1.0-6.0	1.0-6.0	1.0-3.0	1.0-7.0
**Imaging services – N (%)**						
X-ray only	1 (1.8)	2 (4.1)	94 (4.6)	106 (5.2)	3 (0.8)	8 (2.3)
Advanced imaging only	3 (5.4)	1 (2.0)	306 (15.1)	417 (20.6)	14 (3.5)	30 (8.5)
X-ray 1st	0	0	27 (1.3)	11 (0.5)	0	1 (0.3)
Advanced imaging 1st	0	0	36 (1.8)	54 (2.7)	1 (0.3)	0
X-ray and Advanced imaging (on index)	0	0	1 (0.0)	1 (0.0)	3 (0.8)	0
No imaging service	52 (92.9)	46 (93.9)	1564 (77.1)	1439 (71.0)	376 (94.7)	313 (88.9)

(report generated on 23JUL2024)
created on 07292024 using program 2_Aim2_20231116.sas

Table SA6. Healthcare resource utilization for non-specific shoulder diagnosis based on provider type and location

	Non-Specific Shoulder Diagnosis					
	Emergency Department		Primary Care		Specialty Care	
Total (N=181,946)	Physician (N=3865)	Non-Physician (N=1583)	Physician (N=66,471)	Non-Physician (N=80,215)	Physician (N=6131)	Non-Physician (N=23,681)
**Shoulder encounters per patient**						
Mean (SD)	1.74 (2.07)	1.93 (2.28)	2.71 (3.20)	2.70 (3.19)	2.53 (3.13)	3.51 (3.64)
Median (Q1, Q3)	1.0 (1.0, 2.0)	1.0 (1.0, 2.0)	1.0 (1.0, 3.0)	1.0 (1.0, 3.0)	1.0 (1.0, 3.0)	2.0 (1.0, 5.0)
Range	1.0-25.0	1.0-26.0	1.0-35.0	1.0-39.0	1.0-31.0	1.0-39.0
**NSAIDS – N (%)**	349 (9.0)	97 (6.1)	6586 (9.9)	6430 (8.0)	560 (9.1)	1559 (6.6)
Mean (SD)	5.28 (3.30)	5.27 (2.75)	5.33 (3.20)	5.20 (3.14)	4.93 (3.10)	4.82 (3.14)
Median (Q1, Q3)	5.0 (3.0, 7.0)	5.0 (3.0, 7.0)	5.0 (3.0, 7.0)	5.0 (3.0, 7.0)	4.0 (2.0, 7.0)	4.0 (2.0, 7.0)
Range	1.0-19.0	1.0-13.0	1.0-25.0	1.0-22.0	1.0-17.0	1.0-23.0
**Analgesics – N (%)**	183 (4.7)	48 (3.0)	3358 (5.1)	3239 (4.0)	251 (4.1)	762 (3.2)
Mean (SD)	1.77 (1.13)	1.92 (1.38)	1.92 (1.46)	1.88 (1.35)	1.90 (1.36)	1.86 (1.25)
Median (Q1, Q3)	1.0 (1.0, 2.0)	1.5 (1.0, 2.5)	1.0 (1.0, 2.0)	1.0 (1.0, 2.0)	1.0 (1.0, 2.0)	1.0 (1.0, 2.0)
Range	1.0-6.0	1.0-9.0	1.0-20.0	1.0-19.0	1.0-9.0	1.0-12.0
**Muscle relaxers – N (%)**	261 (6.8)	72 (4.5)	4887 (7.4)	4775 (6.0)	396 (6.5)	1146 (4.8)
Mean (SD)	2.36 (1.69)	2.50 (2.10)	2.42 (1.75)	2.47 (1.87)	2.42 (1.88)	2.34 (1.70)
Median (Q1, Q3)	2.0 (1.0, 3.0)	2.0 (1.0, 3.0)	2.0 (1.0, 3.0)	2.0 (1.0, 3.0)	2.0 (1.0, 3.0)	2.0 (1.0, 3.0)
Range	1.0-11.0	1.0-12.0	1.0-17.0	1.0-18.0	1.0-17.0	1.0-14.0
**Opioids – N (%)**	277 (7.2)	83 (5.2)	5310 (8.0)	5134 (6.4)	467 (7.6)	1283 (5.4)
Mean (SD)	3.81 (2.88)	3.65 (3.03)	3.69 (3.15)	3.71 (3.18)	3.40 (2.66)	3.65 (3.14)
Median (Q1, Q3)	3.0 (2.0, 5.0)	3.0 (2.0, 4.0)	3.0 (1.0, 5.0)	3.0 (1.0, 5.0)	3.0 (1.0, 5.0)	3.0 (1.0, 5.0)
Range	1.0-17.0	1.0-16.0	1.0-33.0	1.0-35.0	1.0-17.0	1.0-21.0
**Benzodiazepine – N (%)**	112 (2.9)	31 (2.0)	2247 (3.4)	2205 (2.7)	195 (3.2)	522 (2.2)
Mean (SD)	2.93 (3.11)	2.26 (1.79)	2.19 (1.70)	2.21 (1.79)	2.31 (1.70)	2.33 (1.83)
Median (Q1, Q3)	2.0 (1.0, 3.0)	1.0 (1.0, 3.0)	2.0 (1.0, 3.0)	2.0 (1.0, 3.0)	2.0 (1.0, 3.0)	2.0 (1.0, 3.0)
Range	1.0-21.0	1.0-8.0	1.0-13.0	1.0-15.0	1.0-8.0	1.0-10.0
**Opioids and Benzodiazapines – N (%)**	96 (2.5)	29 (1.8)	1996 (3.0)	1931 (2.4)	178 (2.9)	459 (1.9)
**Steroid joint injections – N (%)**	41 (1.1)	12 (0.8)	1961 (3.0)	1605 (2.0)	731 (11.9)	577 (2.4)
Mean (SD)	1.05 (0.22)	1.17 (0.58)	1.04 (0.19)	1.04 (0.21)	1.07 (0.28)	1.06 (0.35)
Median (Q1, Q3)	1.0 (1.0, 1.0)	1.0 (1.0, 1.0)	1.0 (1.0, 1.0)	1.0 (1.0, 1.0)	1.0 (1.0, 1.0)	1.0 (1.0, 1.0)
Range	1.0-2.0	1.0-3.0	1.0-3.0	1.0-3.0	1.0-3.0	1.0-7.0
**Physical therapy – N (%)**	323 (8.4)	200 (12.6)	18,545 (27.9)	23,296 (29.0)	1084 (17.7)	18876 (79.7)
Mean (SD)	3.47 (3.20)	3.28 (2.97)	4.10 (3.75)	3.90 (3.70)	4.01 (3.87)	3.29 (3.42)
Median (Q1, Q3)	2.0 (1.0, 5.0)	2.0 (1.0, 4.0)	3.0 (1.0, 6.0)	2.0 (1.0, 5.0)	2.0 (1.0, 6.0)	2.0 (1.0, 4.0)
Range	1.0-19.0	1.0-16.0	1.0-31.0	1.0-37.0	1.0-24.0	1.0-28.0

Non-Specific Shoulder Diagnosis						
	Emergency Department		Primary Care		Specialty Care	
Total (N=181,946)	Physician (N=3865)	Non-Physician (N=1583)	Physician (N=66,471)	Non-Physician (N=80,215)	Physician (N=6131)	Non-Physician (N=23,681)
**Passive treatments – N (%)**	108 (2.8)	59 (3.7)	6819 (10.3)	7706 (9.6)	671 (10.9)	5649 (23.9)
Mean (SD)	4.06 (3.72)	3.34 (2.46)	3.97 (3.45)	3.93 (3.45)	3.31 (3.07)	3.27 (2.83)
Median (Q1, Q3)	3.0 (1.0, 5.0)	3.0 (1.0, 5.0)	3.0 (1.0, 5.0)	3.0 (1.0, 5.0)	2.0 (1.0, 5.0)	2.0 (1.0, 4.0)
Range	1.0-21.0	1.0-12.0	1.0-31.0	1.0-29.0	1.0-20.0	1.0-23.0
**Exercise Therapy – N (%)**	331 (8.6)	192 (12.1)	17,895 (26.9)	22,480 (28.0)	1299 (21.2)	16,248 (68.6)
Mean (SD)	3.80 (3.63)	3.66 (3.33)	4.11 (3.69)	4.02 (3.68)	4.05 (3.64)	3.33 (3.31)
Median (Q1, Q3)	3.0 (1.0, 5.0)	3.0 (1.0, 5.0)	3.0 (1.0, 6.0)	3.0 (1.0, 6.0)	3.0 (1.0, 6.0)	2.0 (1.0, 5.0)
Range	1.0-22.0	1.0-20.0	1.0-29.0	1.0-37.0	1.0-22.0	1.0-35.0
**Manual therapy – N (%)**	161 (4.2)	90 (5.7)	9528 (14.3)	10962 (13.7)	523 (8.5)	9549 (40.3)
Mean (SD)	2.99 (3.25)	2.62 (2.25)	3.42 (3.44)	3.33 (3.36)	2.89 (2.82)	2.45 (2.40)
Median (Q1, Q3)	2.0 (1.0, 3.0)	2.0 (1.0, 4.0)	2.0 (1.0, 4.0)	2.0 (1.0, 4.0)	2.0 (1.0, 4.0)	1.0 (1.0, 3.0)
Range	1.0-21.0	1.0-14.0	1.0-28.0	1.0-30.0	1.0-18.0	1.0-27.0
**Acupuncture – N (%)**	15 (0.4)	5 (0.3)	502 (0.8)	315 (0.4)	256 (4.2)	215 (0.9)
Mean (SD)	1.33 (0.90)	1.40 (0.89)	1.43 (1.12)	1.40 (0.88)	1.48 (1.13)	1.70 (1.28)
Median (Q1, Q3)	1.0 (1.0, 1.0)	1.0 (1.0, 1.0)	1.0 (1.0, 1.0)	1.0 (1.0, 1.0)	1.0 (1.0, 2.0)	1.0 (1.0, 2.0)
Range	1.0-4.0	1.0-3.0	1.0-13.0	1.0-6.0	1.0-10.0	1.0-8.0
**Radiographs – N (%)**	33 (0.9)	14 (0.9)	508 (0.8)	531 (0.7)	21 (0.3)	55 (0.2)
Mean (SD)	1.03 (0.17)	1.00 (0.00)	1.18 (0.41)	1.15 (0.36)	1.24 (0.54)	1.15 (0.36)
Median (Q1, Q3)	1.0 (1.0, 1.0)	1.0 (1.0, 1.0)	1.0 (1.0, 1.0)	1.0 (1.0, 1.0)	1.0 (1.0, 1.0)	1.0 (1.0, 1.0)
Range	1.0-2.0	1.0-1.0	1.0-3.0	1.0-3.0	1.0-3.0	1.0-2.0
**Magnetic Resonance Imaging – N (%)**	18 (0.5)	7 (0.4)	1135 (1.7)	1261 (1.6)	52 (0.8)	145 (0.6)
Mean (SD)	1.28 (0.75)	1.00 (0.00)	1.37 (0.51)	1.42 (0.52)	1.25 (0.44)	1.37 (0.51)
Median (Q1, Q3)	1.0 (1.0, 1.0)	1.0 (1.0, 1.0)	1.0 (1.0, 2.0)	1.0 (1.0, 2.0)	1.0 (1.0, 1.5)	1.0 (1.0, 2.0)
Range	1.0-4.0	1.0-1.0	1.0-4.0	1.0-4.0	1.0-2.0	1.0-3.0
**Arthrogram – N (%)**	4 (0.1)	0 (0.0)	108 (0.2)	119 (0.1)	8 (0.1)	24 (0.1)
Mean (SD)	1.00 (0.00)		1.14 (0.35)	1.13 (0.34)	1.13 (0.35)	1.25 (0.44)
Median	1.0 (1.0, 1.0)	.	1.0 (1.0, 1.0)	1.0 (1.0, 1.0)	1.0 (1.0, 1.0)	1.0 (1.0, 1.5)
Range	1.0-1.0		1.0-2.0	1.0-2.0	1.0-2.0	1.0-2.0
**Computed Tomography scans – N (%)**	0	0	6 (0.0)	15 (0.0)	0	0
Mean (SD)			1.50 (0.55)	1.47 (0.52)		
Median (Q1, Q3)	.	.	1.5 (1.0, 2.0)	1.0 (1.0, 2.0)	.	.
Range			1.0-2.0	1.0-2.0		

Non-Specific Shoulder Diagnosis						
Total (N=181,946)	Emergency Department		Primary Care		Specialty Care	
	Physician (N=3865)	Non-Physician (N=1583)	Physician (N=66,471)	Non-Physician (N=80,215)	Physician (N=6131)	Non-Physician (N=23,681)
**Advanced imaging (MRIs, arthrograms, CT scans) – N (%)**	20 (0.5)	7 (0.4)	1144 (1.7)	1285 (1.6)	54 (0.9)	147 (0.6)
Mean (SD)	1.35 (0.93)	1.00 (0.00)	1.47 (0.68)	1.52 (0.67)	1.37 (0.62)	1.56 (0.77)
Median (Q1, Q3)	1.0 (1.0, 1.0)	1.0 (1.0, 1.0)	1.0 (1.0, 2.0)	1.0 (1.0, 2.0)	1.0 (1.0, 2.0)	1.0 (1.0, 2.0)
Range	1.0-5.0	1.0-1.0	1.0-6.0	1.0-4.0	1.0-4.0	1.0-4.0
**Imaging services – N (%)**						
X-ray only	33 (0.9)	13 (0.8)	416 (0.6)	422 (0.5)	18 (0.3)	45 (0.2)
Advanced imaging only	20 (0.5)	6 (0.4)	1052 (1.6)	1176 (1.5)	51 (0.8)	137 (0.6)
X-ray 1st	0	1 (0.1)	39 (0.1)	42 (0.1)	2 (0.0)	5 (0.0)
Advanced imaging 1st	0	0	41 (0.1)	58 (0.1)	0	3 (0.0)
X-ray and Advanced imaging (on index)	0	0	12 (0.0)	9 (0.0)	1 (0.0)	2 (0.0)
No imaging service	3812 (98.6)	1563 (98.7)	64911 (97.7)	78508 (97.9)	6059 (98.8)	23489 (99.2)

(report generated on 23JUL2024)
created on 07292024 using program 2_Aim2_20231116.sas

Table SA7. Healthcare resource utilization for cases with more than one specific shoulder diagnosis, based on provider type and location

	More than 1 specific shoulder diagnosis					
	Emergency Department		Primary Care		Specialty Care	
Total (N=9090)	Physician (N=164)	Non-Physician (N=99)	Physician (N=3773)	Non-Physician (N=3528)	Physician (N=816)	Non-Physician (N=710)
**Shoulder encounters per patient**						
Mean (SD)	7.62 (5.08)	6.70 (4.83)	8.54 (5.59)	8.57 (5.64)	6.69 (5.56)	7.55 (5.38)
Median (Q1, Q3)	6.0 (4.0, 9.0)	5.0 (3.0, 10.0)	7.0 (4.0, 12.0)	7.0 (4.0, 11.0)	5.0 (3.0, 9.0)	6.0 (3.0, 10.0)
Range	1.0-26.0	1.0-22.0	1.0-39.0	1.0-40.0	1.0-32.0	1.0-39.0
**NSAIDS – N (%)**	25 (15.2)	10 (10.1)	559 (14.8)	456 (12.9)	101 (12.4)	83 (11.7)
Mean (SD)	5.04 (3.18)	4.60 (3.60)	5.33 (3.28)	5.15 (3.15)	3.95 (2.75)	4.66 (3.46)
Median (Q1, Q3)	5.0 (3.0, 7.0)	3.5 (3.0, 7.0)	5.0 (3.0, 7.0)	5.0 (3.0, 7.0)	3.0 (2.0, 5.0)	4.0 (2.0, 7.0)
Range	1.0-12.0	1.0-13.0	1.0-20.0	1.0-17.0	1.0-13.0	1.0-20.0
**Analgesics – N (%)**	11 (6.7)	3 (3.0)	279 (7.4)	229 (6.5)	54 (6.6)	47 (6.6)
Mean (SD)	2.55 (1.69)	1.67 (1.15)	1.86 (1.26)	2.09 (1.71)	1.59 (0.98)	1.45 (0.77)
Median (Q1, Q3)	2.0 (1.0, 4.0)	1.0 (1.0, 3.0)	1.0 (1.0, 2.0)	1.0 (1.0, 3.0)	1.0 (1.0, 2.0)	1.0 (1.0, 2.0)
Range	1.0-5.0	1.0-3.0	1.0-9.0	1.0-11.0	1.0-5.0	1.0-4.0
**Muscle relaxers – N (%)**	22 (13.4)	7 (7.1)	433 (11.5)	318 (9.0)	63 (7.7)	55 (7.7)
Mean (SD)	2.68 (1.70)	2.14 (1.21)	2.54 (1.77)	2.50 (1.72)	2.62 (2.04)	2.24 (1.54)
Median (Q1, Q3)	2.0 (1.0, 4.0)	2.0 (1.0, 3.0)	2.0 (1.0, 3.0)	2.0 (1.0, 3.0)	2.0 (1.0, 3.0)	2.0 (1.0, 3.0)
Range	1.0-7.0	1.0-4.0	1.0-11.0	1.0-10.0	1.0-11.0	1.0-8.0
**Opioids – N (%)**	20 (12.2)	7 (7.1)	458 (12.1)	345 (9.8)	76 (9.3)	68 (9.6)
Mean (SD)	4.90 (4.61)	4.43 (2.30)	3.53 (2.87)	3.84 (3.37)	3.89 (3.31)	3.40 (2.42)
Median (Q1, Q3)	3.0 (2.0, 7.0)	4.0 (2.0, 7.0)	3.0 (2.0, 5.0)	3.0 (1.0, 5.0)	3.0 (1.5, 5.0)	3.0 (1.0, 5.0)
Range	1.0-20.0	2.0-8.0	1.0-26.0	1.0-24.0	1.0-18.0	1.0-12.0
**Benzodiazepine – N (%)**	10 (6.1)	3 (3.0)	196 (5.2)	154 (4.4)	35 (4.3)	26 (3.7)
Mean (SD)	2.90 (2.42)	2.67 (0.58)	2.20 (1.59)	1.94 (1.30)	2.31 (1.62)	2.27 (1.87)
Median (Q1, Q3)	2.0 (1.0, 4.0)	3.0 (2.0, 3.0)	2.0 (1.0, 3.0)	1.0 (1.0, 3.0)	2.0 (1.0, 3.0)	1.5 (1.0, 3.0)
Range	1.0-8.0	2.0-3.0	1.0-9.0	1.0-7.0	1.0-7.0	1.0-8.0
**Opioids and Benzodiazapines – N (%)**	9 (5.5)	2 (2.0)	177 (4.7)	135 (3.8)	28 (3.4)	23 (3.2)
**Steroid joint injections – N (%)**	42 (25.6)	25 (25.3)	1200 (31.8)	1149 (32.6)	239 (29.3)	257 (36.2)
Mean (SD)	1.14 (0.35)	1.04 (0.20)	1.14 (0.38)	1.12 (0.35)	1.18 (0.45)	1.13 (0.37)
Median (Q1, Q3)	1.0 (1.0, 1.0)	1.0 (1.0, 1.0)	1.0 (1.0, 1.0)	1.0 (1.0, 1.0)	1.0 (1.0, 1.0)	1.0 (1.0, 1.0)
Range	1.0-2.0	1.0-2.0	1.0-4.0	1.0-4.0	1.0-4.0	1.0-3.0
**Physical therapy – N (%)**	86 (52.4)	52 (52.5)	2216 (58.7)	2010 (57.0)	390 (47.8)	509 (71.7)
Mean (SD)	4.14 (3.82)	5.33 (3.87)	5.64 (4.73)	5.60 (4.66)	5.76 (4.93)	5.29 (4.60)
Median (Q1, Q3)	3.0 (2.0, 5.0)	5.0 (2.0, 7.5)	4.0 (2.0, 8.0)	4.0 (2.0, 8.0)	4.0 (2.0, 9.0)	4.0 (2.0, 8.0)
Range	1.0-18.0	1.0-16.0	1.0-35.0	1.0-30.0	1.0-29.0	1.0-26.0

Total (N=9090)	More than 1 specific shoulder diagnosis					
	Emergency Department		Primary Care		Specialty Care	
	Physician (N=164)	Non-Physician (N=99)	Physician (N=3773)	Non-Physician (N=3528)	Physician (N=816)	Non-Physician (N=710)
**Passive treatments – N (%)**	49 (29.9)	24 (24.2)	1206 (32.0)	1085 (30.8)	222 (27.2)	241 (33.9)
Mean (SD)	4.10 (3.29)	3.46 (3.04)	5.31 (4.42)	5.25 (4.57)	5.13 (4.57)	4.55 (3.78)
Median (Q1, Q3)	3.0 (1.0, 6.0)	3.0 (1.5, 4.0)	4.0 (2.0, 7.0)	4.0 (2.0, 7.0)	4.0 (1.0, 8.0)	4.0 (2.0, 6.0)
Range	1.0-14.0	1.0-14.0	1.0-35.0	1.0-29.0	1.0-25.0	1.0-31.0
**Exercise Therapy – N (%)**	100 (61.0)	53 (53.5)	2312 (61.3)	2098 (59.5)	405 (49.6)	474 (66.8)
Mean (SD)	4.54 (4.05)	4.75 (3.65)	5.92 (4.69)	5.74 (4.72)	5.98 (4.75)	5.08 (4.37)
Median (Q1, Q3)	3.0 (2.0, 6.0)	4.0 (2.0, 7.0)	5.0 (2.0, 9.0)	5.0 (2.0, 8.0)	5.0 (2.0, 9.0)	4.0 (2.0, 7.0)
Range	1.0-17.0	1.0-16.0	1.0-32.0	1.0-31.0	1.0-27.0	1.0-27.0
**Manual therapy – N (%)**	60 (36.6)	26 (26.3)	1465 (38.8)	1262 (35.8)	216 (26.5)	258 (36.3)
Mean (SD)	3.28 (3.14)	4.12 (3.66)	5.32 (4.50)	5.21 (4.59)	4.50 (4.20)	3.59 (3.55)
Median (Q1, Q3)	2.0 (1.0, 4.0)	2.0 (1.0, 6.0)	4.0 (2.0, 8.0)	4.0 (2.0, 8.0)	3.0 (1.0, 6.0)	2.0 (1.0, 5.0)
Range	1.0-13.0	1.0-16.0	1.0-32.0	1.0-28.0	1.0-23.0	1.0-18.0
**Acupuncture – N (%)**	2 (1.2)	1 (1.0)	25 (0.7)	20 (0.6)	7 (0.9)	9 (1.3)
Mean (SD)	2.50 (2.12)	1.00 (.)	1.68 (1.44)	1.85 (1.42)	3.14 (4.41)	2.78 (3.60)
Median (Q1, Q3)	2.5 (1.0, 4.0)	1.0 (1.0, 1.0)	1.0 (1.0, 2.0)	1.0 (1.0, 2.0)	1.0 (1.0, 3.0)	1.0 (1.0, 2.0)
Range	1.0-4.0	1.0-1.0	1.0-7.0	1.0-5.0	1.0-13.0	1.0-12.0
**Radiographs – N (%)**	23 (14.0)	15 (15.2)	724 (19.2)	716 (20.3)	29 (3.6)	44 (6.2)
Mean (SD)	1.30 (0.63)	1.13 (0.35)	1.18 (0.46)	1.17 (0.43)	1.21 (0.41)	1.34 (0.53)
Median (Q1, Q3)	1.0 (1.0, 1.0)	1.0 (1.0, 1.0)	1.0 (1.0, 1.0)	1.0 (1.0, 1.0)	1.0 (1.0, 1.0)	1.0 (1.0, 2.0)
Range	1.0-3.0	1.0-2.0	1.0-5.0	1.0-4.0	1.0-2.0	1.0-3.0
**Magnetic Resonance Imaging – N (%)**	31 (18.9)	15 (15.2)	1358 (36.0)	1410 (40.0)	71 (8.7)	119 (16.8)
Mean (SD)	1.39 (0.67)	1.20 (0.41)	1.51 (0.60)	1.55 (0.58)	1.45 (0.60)	1.45 (0.53)
Median (Q1, Q3)	1.0 (1.0, 2.0)	1.0 (1.0, 1.0)	1.0 (1.0, 2.0)	2.0 (1.0, 2.0)	1.0 (1.0, 2.0)	1.0 (1.0, 2.0)
Range	1.0-4.0	1.0-2.0	1.0-5.0	1.0-4.0	1.0-4.0	1.0-3.0
**Arthrogram – N (%)**	2 (1.2)	1 (1.0)	128 (3.4)	117 (3.3)	11 (1.3)	18 (2.5)
Mean (SD)	1.00 (0.00)	1.00 (.)	1.21 (0.41)	1.31 (0.56)	1.00 (0.00)	1.33 (0.49)
Median (Q1, Q3)	1.0 (1.0, 1.0)	1.0 (1.0, 1.0)	1.0 (1.0, 1.0)	1.0 (1.0, 2.0)	1.0 (1.0, 1.0)	1.0 (1.0, 2.0)
Range	1.0-1.0	1.0-1.0	1.0-2.0	1.0-4.0	1.0-1.0	1.0-2.0
**Computed Tomography scans – N (%)**	1 (0.6)	0	18 (0.5)	11 (0.3)	1 (0.1)	1 (0.1)
Mean (SD)	2.00 (.)		1.67 (0.59)	1.36 (0.50)	1.00 (.)	1.00 (.)
Median (Q1, Q3)	2.0 (2.0-2.0)	.	2.0 (1.0-2.0)	1.0 (1.0-2.0)	1.0 (1.0-1.0)	1.0 (1.0-1.0)
Range	2.0-2.0		1.0-3.0	1.0-2.0	1.0-1.0	1.0-1.0

More than 1 specific shoulder diagnosis						
Total (N=9090)	Emergency Department		Primary Care		Specialty Care	
	Physician (N=164)	Non-Physician (N=99)	Physician (N=3773)	Non-Physician (N=3528)	Physician (N=816)	Non-Physician (N=710)
**Advanced imaging (MRIs, arthrograms, CT scans) – N (%)**	32 (19.5)	15 (15.2)	1371 (36.3)	1422 (40.3)	73 (8.9)	121 (17.0)
Mean (SD)	1.47 (0.72)	1.27 (0.59)	1.63 (0.78)	1.66 (0.77)	1.58 (0.76)	1.64 (0.85)
Median (Q1, Q3)	1.0 (1.0, 2.0)	1.0 (1.0, 1.0)	1.0 (1.0, 2.0)	2.0 (1.0, 2.0)	1.0 (1.0, 2.0)	1.0 (1.0, 2.0)
Range	1.0-4.0	1.0-3.0	1.0-6.0	1.0-8.0	1.0-4.0	1.0-4.0
**Imaging services – N (%)**						
X-ray only	12 (7.3)	12 (12.1)	259 (6.9)	232 (6.6)	13 (1.6)	24 (3.4)
Advanced imaging only	21 (12.8)	12 (12.1)	906 (24.0)	938 (26.6)	57 (7.0)	101 (14.2)
X-ray 1st	5 (3.0)	2 (2.0)	207 (5.5)	153 (4.3)	5 (0.6)	12 (1.7)
Advanced imaging 1st	5 (3.0)	1 (1.0)	246 (6.5)	316 (9.0)	2 (0.2)	6 (0.8)
X-ray and Advanced imaging (on index)	1 (0.6)	0	12 (0.3)	15 (0.4)	9 (1.1)	2 (0.3)
No imaging service	120 (73.2)	72 (72.7)	2143 (56.8)	1874 (53.1)	730 (89.5)	565 (79.6)

(report generated on 23JUL2024)
created on 07292024 using program 2_Aim2_20231116.sas

Figure SA1. Proportion of case within each diagnostic subgroup across index provider types

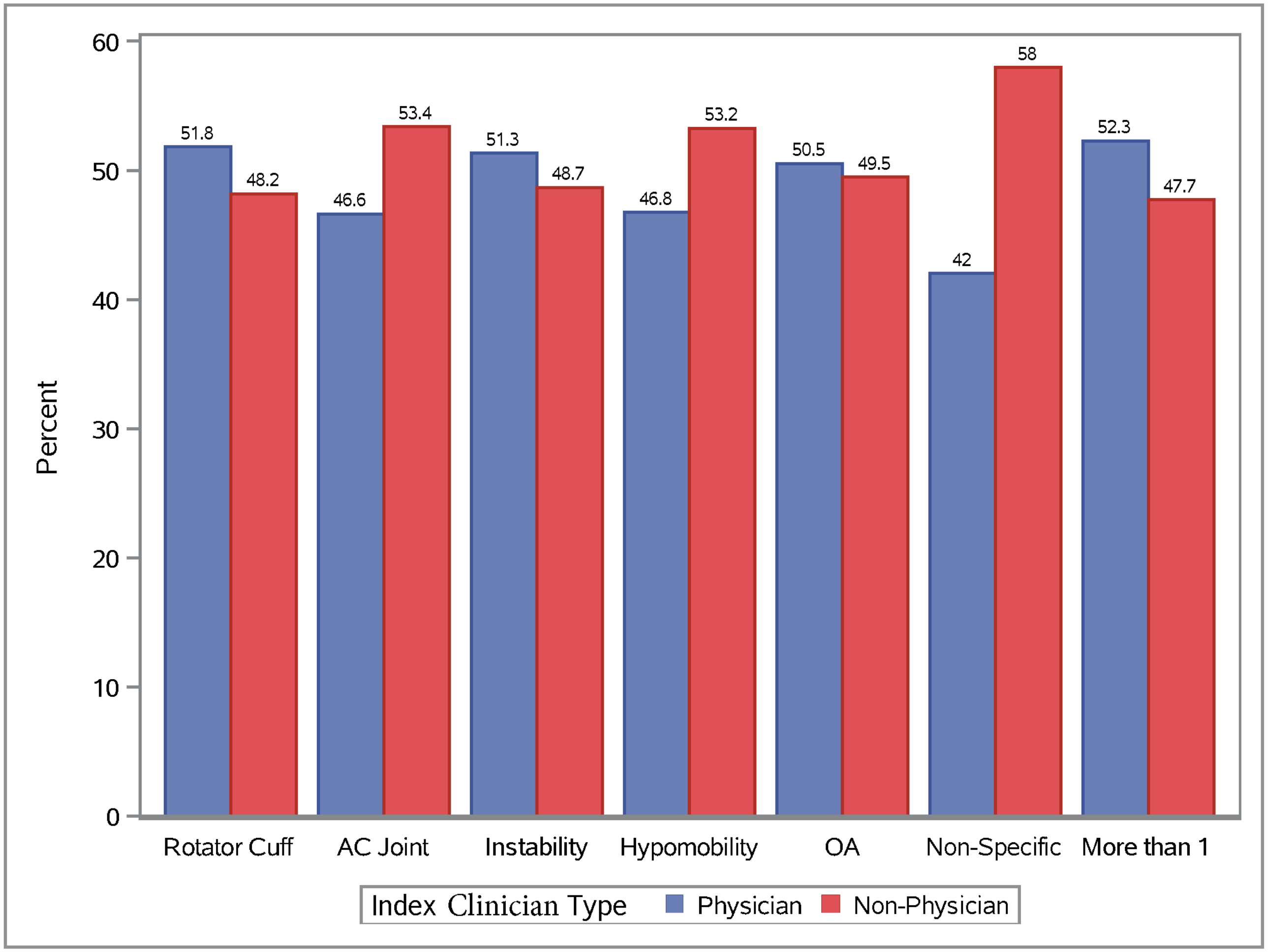

Note: AC = Acromioclavicular; Instability includes closed dislocation; Hypomobility includes adhesive capsulitis; OA = Osteoarthritis; Non-Specific = non-specific shoulder pain (no specific diagnosis rendered); More than 1 = more than 1 specific shoulder diagnostic category

Figure SA2. Mean number of patient visits for each condition, by clinician type.

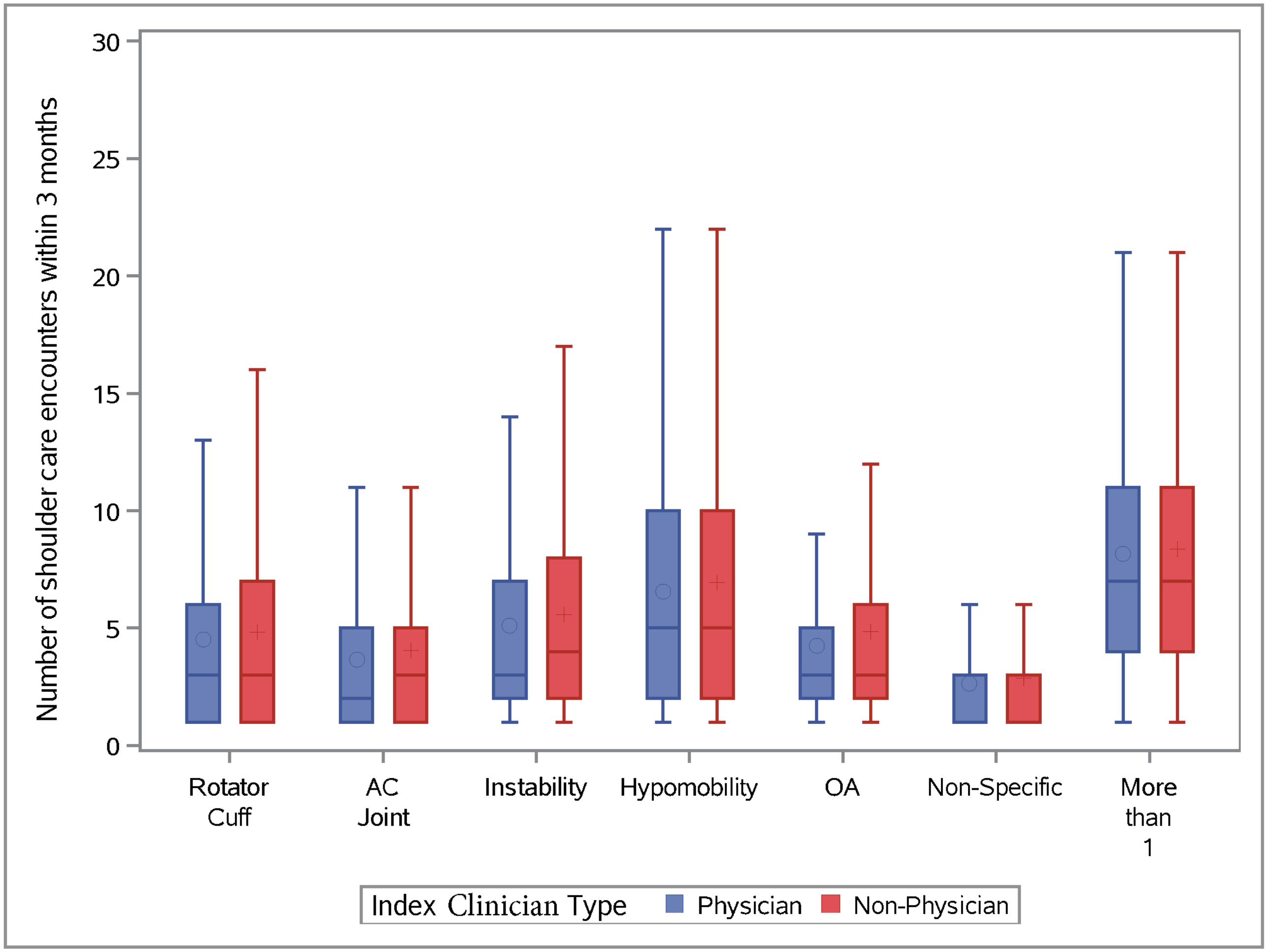

Note: AC = Acromioclavicular; Instability includes closed dislocation; Hypomobility includes adhesive capsulitis; OA = Osteoarthritis; Non-Specific = non-specific shoulder pain (no specific diagnosis rendered); More than 1 = more than 1 specific shoulder diagnostic category

Figure SA3. Proportion of patients receiving pharmacological care by clinician type, specific shoulder diagnostic group within setting

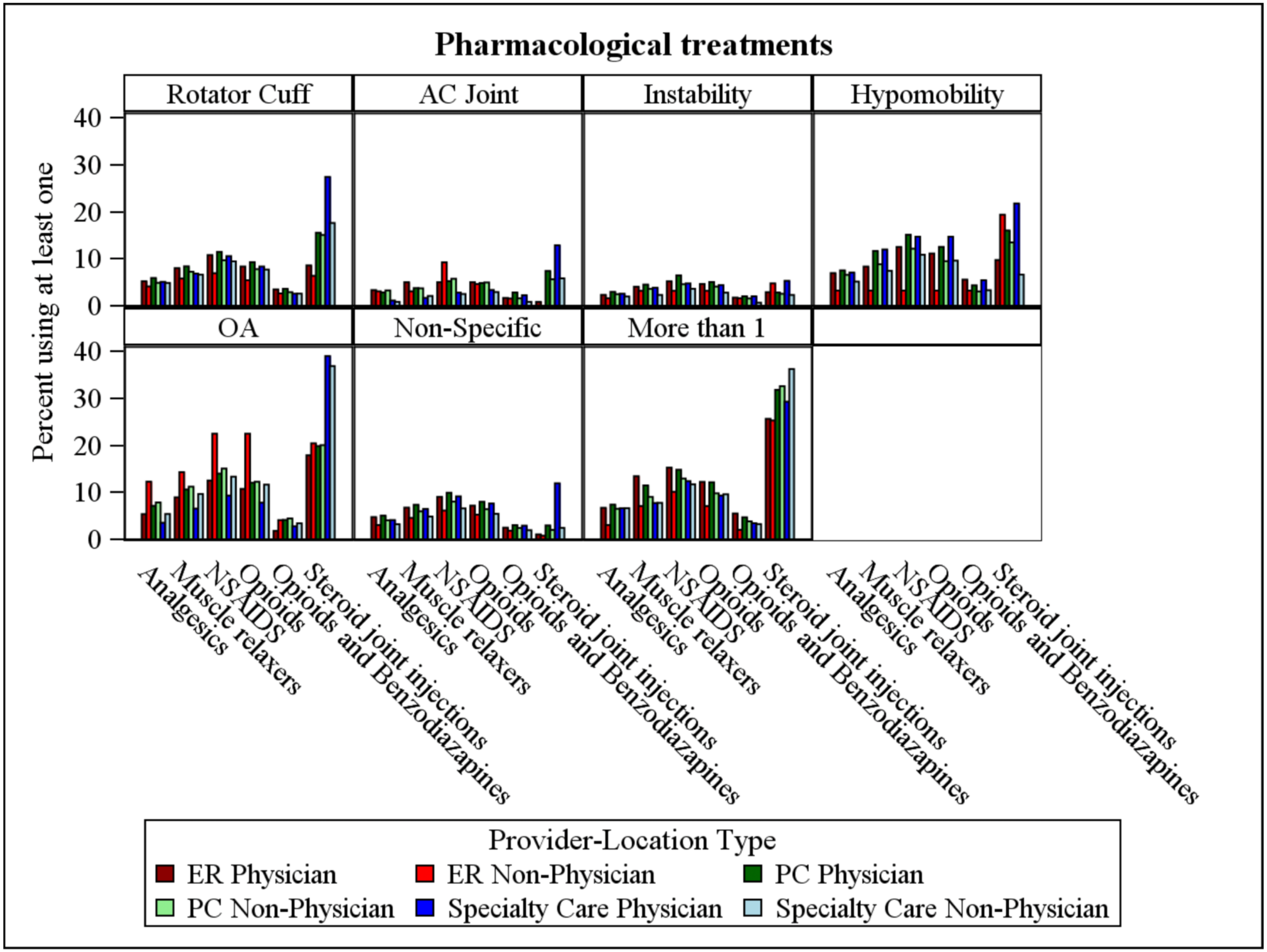

Note: AC = Acromioclavicular; Instability includes closed dislocation; Hypomobility includes adhesive capsulitis; OA = Osteoarthritis; Non-Specific = non-specific shoulder pain (no specific diagnosis rendered); More than 1 = more than 1 specific shoulder diagnostic category

Figure SA4. Proportion of patients receiving non-pharmacological care by clinician type, specific shoulder diagnostic group within setting

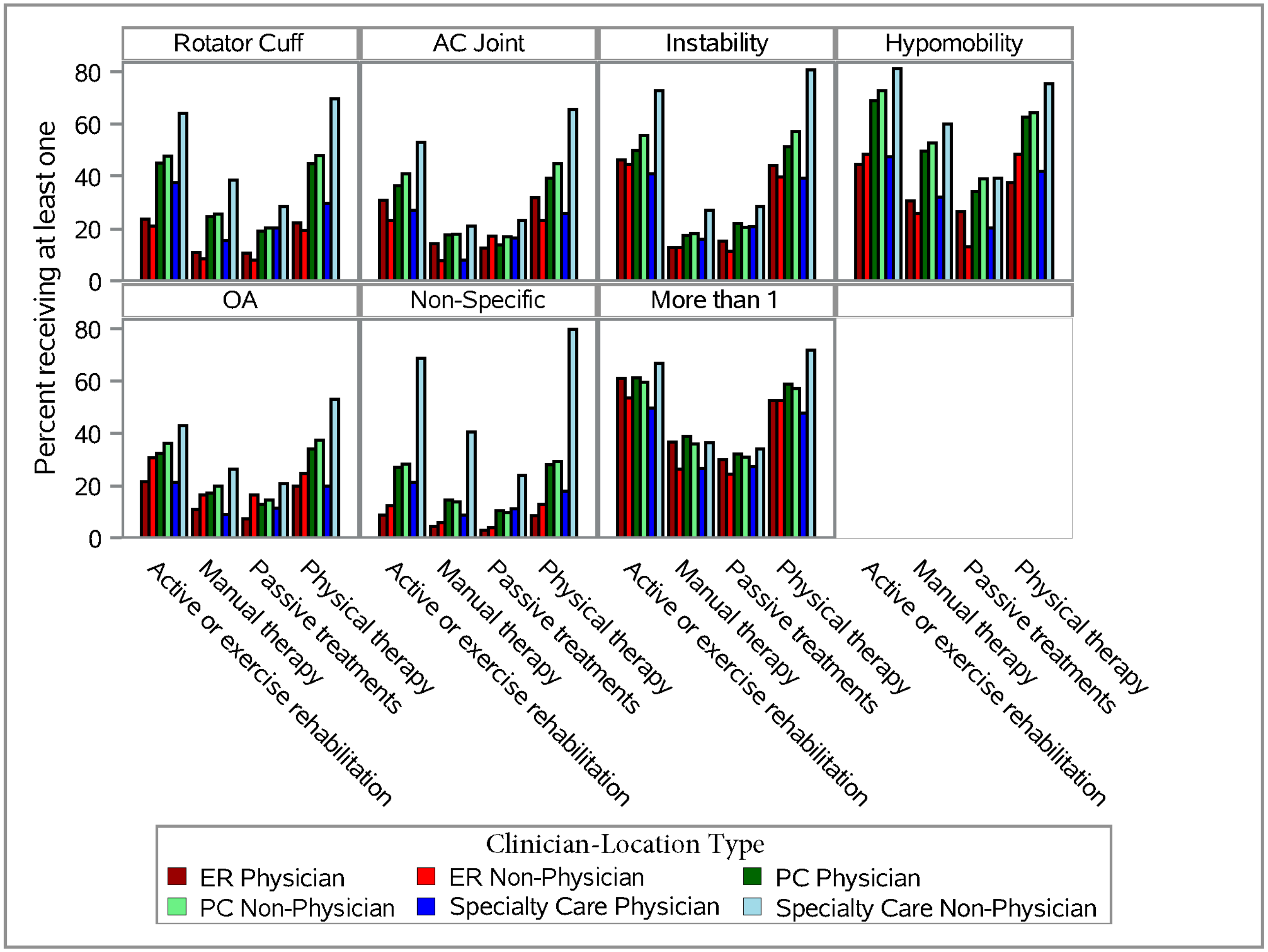

Note: AC = Acromioclavicular; Instability includes closed dislocation; Hypomobility includes adhesive capsulitis; OA = Osteoarthritis; Non-Specific = non-specific shoulder pain (no specific diagnosis rendered); More than 1 = more than 1 specific shoulder diagnostic category

Figure SA5. Proportion of patients receiving diagnostic imaging by clinician type, specific shoulder diagnostic group within setting

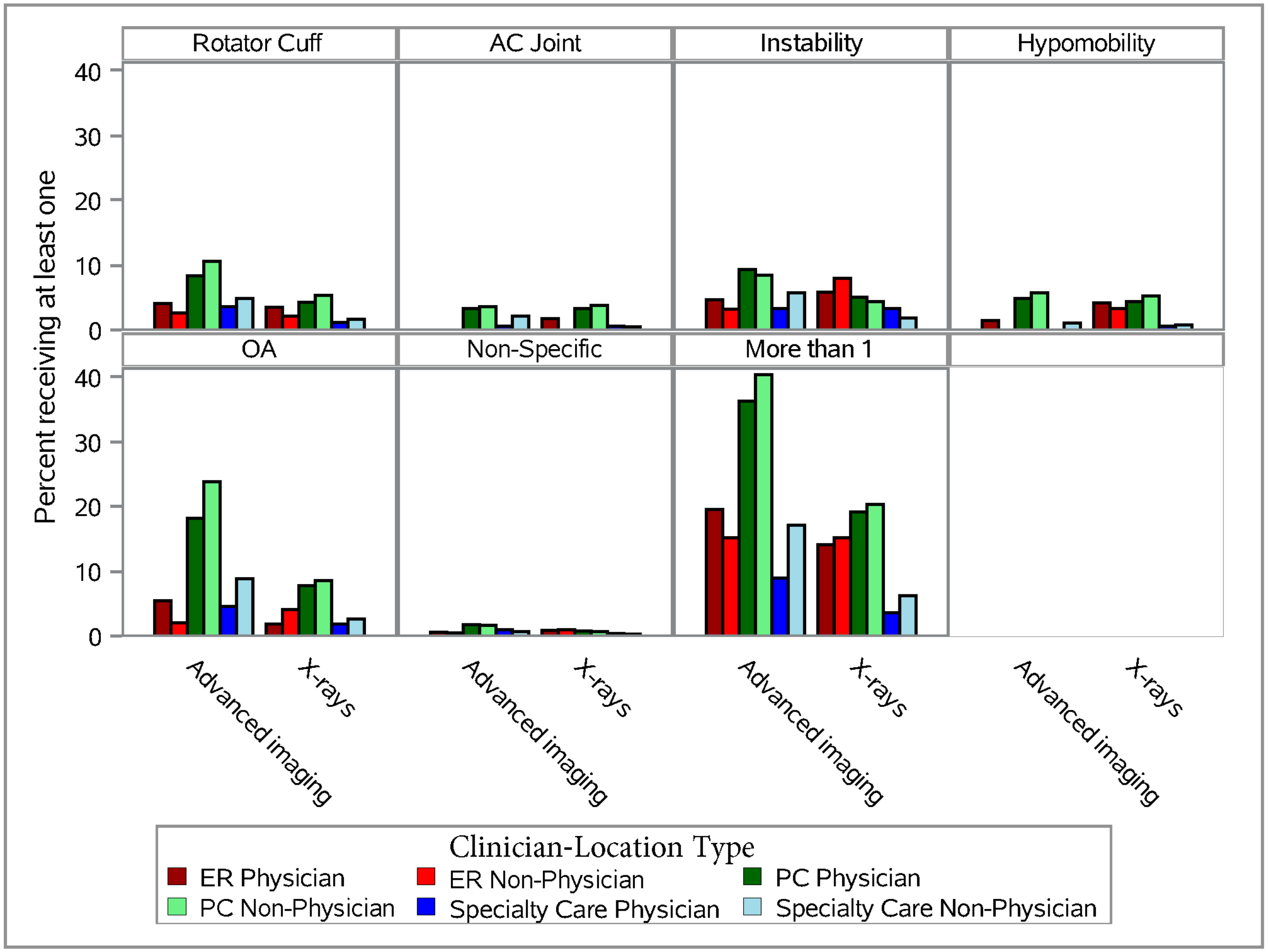

Note: AC = Acromioclavicular; Instability includes closed dislocation; Hypomobility includes adhesive capsulitis; OA = Osteoarthritis; Non-Specific = non-specific shoulder pain (no specific diagnosis rendered); More than 1 = more than 1 specific shoulder diagnostic category

TABLE SA.8: ICD Diagnosis Codes – Rotator Cuff Disorders

ICD9 Code, WITHOUT decimals	ICD9 Code, WITH decimals	Description
	726.1x	"Rotator Cuff Syndrome"
72610	726.10	Tenosynovitis shoulder
72611	726.11	Calcifying tendinitis of shoulder
72612	726.12	Bicipital tenosynovitis
72613	726.13	Partial tear of rotator cuff
72619	726.19	Other specified disorders of bursae and tendons in shoulder region
72761	727.61	Nontraumatic rupture of rotator cuff
8403	840.30	Infraspinatus (muscle) (tendon) sprain
8404	840.4	Rotator cuff (capsule) sprain
8405	840.5	Subscapularis (muscle) sprain
8406	840.6	Supraspinatus (muscle) (tendon) sprain

ICD10 Code, WITHOUT decimals	ICD10 Code, WITH decimals	Description
M62011	M62.011	Separation of muscle (nontraumatic), right shoulder
M62012	M62.012	Separation of muscle (nontraumatic), left shoulder
M62019	M62.019	Separation of muscle (nontraumatic), unspecified shoulder
M62111	M62.111	Other rupture of muscle (nontraumatic), right shoulder
M62112	M62.112	Other rupture of muscle (nontraumatic), left shoulder
M62119	M62.119	Other rupture of muscle (nontraumatic), unspecified shoulder
M71811	M71.811	Other specified bursopathies, right shoulder
M71812	M71.812	Other specified bursopathies, left shoulder
M71819	M71.819	Other specified bursopathies, unspecified shoulder
M75100	M75.100	Unspecified rotator cuff tear or rupture of unspecified shoulder, not specified as traumatic
M75101	M75.101	Unspecified rotator cuff tear or rupture of right shoulder, not specified as traumatic
M75102	M75.102	Unspecified rotator cuff tear or rupture of left shoulder, not specified as traumatic
M75110	M75.110	Incomplete rotator cuff tear or rupture of unspecified shoulder, not specified as traumatic
M75111	M75.111	Incomplete rotator cuff tear or rupture of right shoulder, not specified as traumatic
M75112	M75.112	Incomplete rotator cuff tear or rupture of left shoulder, not specified as traumatic
M75120	M75.120	Complete rotator cuff tear or rupture of unspecified shoulder, not specified as traumatic
M75121	M75.121	Complete rotator cuff tear or rupture of right shoulder, not specified as traumatic
M75122	M75.122	Complete rotator cuff tear or rupture of left shoulder, not specified as traumatic
M7520	M75.20	Bicipital tendinitis, unspecified shoulder
M7521	M75.21	Bicipital tendinitis, right shoulder
M7522	M75.22	Bicipital tendinitis, left shoulder
M7530	M75.30	Calcific tendinitis of unspecified shoulder
M7531	M75.31	Calcific tendinitis of right shoulder
M7532	M75.32	Calcific tendinitis of left shoulder
M7540	M75.40	Impingement syndrome of unspecified shoulder
M7541	M75.41	Impingement syndrome of right shoulder
M7542	M75.42	Impingement syndrome of left shoulder

M7550	M75.50	Bursitis of unspecified shoulder
M7551	M75.51	Bursitis of right shoulder
M7552	M75.52	Bursitis of left shoulder
M7550	M75.50	Bursitis of unspecified shoulder
M7551	M75.51	Bursitis of right shoulder
M7552	M75.52	Bursitis of left shoulder
S43421A	S43.421A	Sprain of right rotator cuff capsule, initial encounter
S43421D	S43.421D	Sprain of right rotator cuff capsule, subsequent encounter
S43421S	S43.421S	Sprain of right rotator cuff capsule, sequela
S43422A	S43.422A	Sprain of left rotator cuff capsule, initial encounter
S43422D	S43.422D	Sprain of left rotator cuff capsule, subsequent encounter
S43422S	S43.422S	Sprain of left rotator cuff capsule, sequela
S43429A	S43.429A	Sprain of unspecified rotator cuff capsule, initial encounter
S43429D	S43.429D	Sprain of unspecified rotator cuff capsule, subsequent encounter
S43429S	S43.429S	Sprain of unspecified rotator cuff capsule, sequela
S46001A		Unspecified injury of muscle(s) and tendon(s) of the rotator cuff of right shoulder, initial encounter
S46001D		Unspecified injury of muscle(s) and tendon(s) of the rotator cuff of right shoulder, subsequent encounter
S46001S		Unspecified injury of muscle(s) and tendon(s) of the rotator cuff of right shoulder, sequela
S46002A		Unspecified injury of muscle(s) and tendon(s) of the rotator cuff of left shoulder, initial encounter
S46002D		Unspecified injury of muscle(s) and tendon(s) of the rotator cuff of left shoulder, subsequent encounter
S46002S		Unspecified injury of muscle(s) and tendon(s) of the rotator cuff of left shoulder, sequela
S46009A		Unspecified injury of muscle(s) and tendon(s) of the rotator cuff of unspecified shoulder, initial encounter
S46009D		Unspecified injury of muscle(s) and tendon(s) of the rotator cuff of unspecified shoulder, subsequent encounter
S46009S		Unspecified injury of muscle(s) and tendon(s) of the rotator cuff of unspecified shoulder, sequela
S46011A		Strain of muscle(s) and tendon(s) of the rotator cuff of right shoulder, initial encounter
S46011D		Strain of muscle(s) and tendon(s) of the rotator cuff of right shoulder, subsequent encounter
S46011S		Strain of muscle(s) and tendon(s) of the rotator cuff of right shoulder, sequela
S46012A		Strain of muscle(s) and tendon(s) of the rotator cuff of left shoulder, initial encounter
S46012D		Strain of muscle(s) and tendon(s) of the rotator cuff of left shoulder, subsequent encounter
S46012S		Strain of muscle(s) and tendon(s) of the rotator cuff of left shoulder, sequela
S46019A		Strain of muscle(s) and tendon(s) of the rotator cuff of unspecified shoulder, initial encounter
S46019D		Strain of muscle(s) and tendon(s) of the rotator cuff of unspecified shoulder, subsequent encounter
S46019S		Strain of muscle(s) and tendon(s) of the rotator cuff of unspecified shoulder, sequela
S46021A		Laceration of muscle(s) and tendon(s) of the rotator cuff of right shoulder, initial encounter
S46021D		Laceration of muscle(s) and tendon(s) of the rotator cuff of right shoulder, subsequent encounter
S46021S		Laceration of muscle(s) and tendon(s) of the rotator cuff of right shoulder, sequela
S46022A		Laceration of muscle(s) and tendon(s) of the rotator cuff of left shoulder, initial encounter
S46022D		Laceration of muscle(s) and tendon(s) of the rotator cuff of left shoulder, subsequent encounter
S46022S		Laceration of muscle(s) and tendon(s) of the rotator cuff of left shoulder, sequela
S46029A		Laceration of muscle(s) and tendon(s) of the rotator cuff of unspecified shoulder, initial encounter
S46029D		Laceration of muscle(s) and tendon(s) of the rotator cuff of unspecified shoulder, subsequent encounter
S46029S		Laceration of muscle(s) and tendon(s) of the rotator cuff of unspecified shoulder, sequela
S46091A		Other injury of muscle(s) and tendon(s) of the rotator cuff of right shoulder, initial encounter

S46091D		Other injury of muscle(s) and tendon(s) of the rotator cuff of right shoulder, subsequent encounter
S46091S		Other injury of muscle(s) and tendon(s) of the rotator cuff of right shoulder, sequela
S46092A		Other injury of muscle(s) and tendon(s) of the rotator cuff of left shoulder, initial encounter
S46092D		Other injury of muscle(s) and tendon(s) of the rotator cuff of left shoulder, subsequent encounter
S46092S		Other injury of muscle(s) and tendon(s) of the rotator cuff of left shoulder, sequela
S46099A		Other injury of muscle(s) and tendon(s) of the rotator cuff of unspecified shoulder, initial encounter
S46099D		Other injury of muscle(s) and tendon(s) of the rotator cuff of unspecified shoulder, subsequent encounter
S46099S		Other injury of muscle(s) and tendon(s) of the rotator cuff of unspecified shoulder, sequela

TABLE SA.9: ICD Diagnosis Codes – Acromioclavicular Joint Dysfunction

ICD10 Code, WITHOUT decimals	ICD10 Code, WITH decimals	Description
S43101A	S43.101A	Unspecified dislocation of right acromioclavicular joint, initial encounter
S43101D	S43.101D	Unspecified dislocation of right acromioclavicular joint, subsequent encounter
S43101S	S43.101S	Unspecified dislocation of right acromioclavicular joint, sequela
S43102A	S43.102A	Unspecified dislocation of left acromioclavicular joint, initial encounter
S43102D	S43.102D	Unspecified dislocation of left acromioclavicular joint, subsequent encounter
S43102S	S43.102S	Unspecified dislocation of left acromioclavicular joint, sequela
S43109A	S43.109A	Unspecified dislocation of unspecified acromioclavicular joint, initial encounter
S43109D	S43.109D	Unspecified dislocation of unspecified acromioclavicular joint, subsequent encounter
S43109S	S43.109S	Unspecified dislocation of unspecified acromioclavicular joint, sequela
S43111A	S43.111A	Subluxation of right acromioclavicular joint, initial encounter
S43111D	S43.111D	Subluxation of right acromioclavicular joint, subsequent encounter
S43111S	S43.111S	Subluxation of right acromioclavicular joint, sequela
S43112A	S43.112A	Subluxation of left acromioclavicular joint, initial encounter
S43112D	S43.112D	Subluxation of left acromioclavicular joint, subsequent encounter
S43112S	S43.112S	Subluxation of left acromioclavicular joint, sequela
S43119A	S43.119A	Subluxation of unspecified acromioclavicular joint, initial encounter
S43119D	S43.119D	Subluxation of unspecified acromioclavicular joint, subsequent encounter
S43119S	S43.119S	Subluxation of unspecified acromioclavicular joint, sequela
S43121A	S43.121A	Dislocation of right acromioclavicular joint, 100%-200% displacement, initial encounter
S43121D	S43.121D	Dislocation of right acromioclavicular joint, 100%-200% displacement, subsequent encounter
S43121S	S43.121S	Dislocation of right acromioclavicular joint, 100%-200% displacement, sequela
S43122A	S43.122A	Dislocation of left acromioclavicular joint, 100%-200% displacement, initial encounter
S43122D	S43.122D	Dislocation of left acromioclavicular joint, 100%-200% displacement, subsequent encounter
S43122S	S43.122S	Dislocation of left acromioclavicular joint, 100%-200% displacement, sequela
S43129A	S43.129A	Dislocation of unspecified acromioclavicular joint, 100%-200% displacement, initial encounter
S43129D	S43.129D	Dislocation of unspecified acromioclavicular joint, 100%-200% displacement, subsequent encounter
S43129S	S43.129S	Dislocation of unspecified acromioclavicular joint, 100%-200% displacement, sequela
S43131A	S43.131A	Dislocation of right acromioclavicular joint, greater than 200% displacement, initial encounter
S43131D	S43.131D	Dislocation of right acromioclavicular joint, greater than 200% displacement, subsequent encounter
S43131S	S43.131S	Dislocation of right acromioclavicular joint, greater than 200% displacement, sequela
S43132A	S43.132A	Dislocation of left acromioclavicular joint, greater than 200% displacement, initial encounter

S43132D	S43.132D	Dislocation of left acromioclavicular joint, greater than 200% displacement, subsequent encounter
S43132S	S43.132S	Dislocation of left acromioclavicular joint, greater than 200% displacement, sequela
S43139A	S43.139A	Dislocation of unspecified acromioclavicular joint, greater than 200% displacement, initial encounter
S43139D	S43.139D	Dislocation of unspecified acromioclavicular joint, greater than 200% displacement, subsequent encounter
S43139S	S43.139S	Dislocation of unspecified acromioclavicular joint, greater than 200% displacement, sequela
S43141A	S43.141A	Inferior dislocation of right acromioclavicular joint, initial encounter
S43141D	S43.141D	Inferior dislocation of right acromioclavicular joint, subsequent encounter
S43141S	S43.141S	Inferior dislocation of right acromioclavicular joint, sequela
S43142A	S43.142A	Inferior dislocation of left acromioclavicular joint, initial encounter
S43142D	S43.142D	Inferior dislocation of left acromioclavicular joint, subsequent encounter
S43142S	S43.142S	Inferior dislocation of left acromioclavicular joint, sequela
S43149A	S43.149A	Inferior dislocation of unspecified acromioclavicular joint, initial encounter
S43149D	S43.149D	Inferior dislocation of unspecified acromioclavicular joint, subsequent encounter
S43149S	S43.149S	Inferior dislocation of unspecified acromioclavicular joint, sequela
S43151A	S43.151A	Posterior dislocation of right acromioclavicular joint, initial encounter
S43151D	S43.151D	Posterior dislocation of right acromioclavicular joint, subsequent encounter
S43151S	S43.151S	Posterior dislocation of right acromioclavicular joint, sequela
S43152A	S43.152A	Posterior dislocation of left acromioclavicular joint, initial encounter
S43152D	S43.152D	Posterior dislocation of left acromioclavicular joint, subsequent encounter
S43152S	S43.152S	Posterior dislocation of left acromioclavicular joint, sequela
S43159A	S43.159A	Posterior dislocation of unspecified acromioclavicular joint, initial encounter
S43159D	S43.159D	Posterior dislocation of unspecified acromioclavicular joint, subsequent encounter
S43159S	S43.159S	Posterior dislocation of unspecified acromioclavicular joint, sequela
S4350XA	S43.50XA	Sprain of unspecified acromioclavicular joint, initial encounter
S4350XD	S43.50XD	Sprain of unspecified acromioclavicular joint, subsequent encounter
S4350XS	S43.50XS	Sprain of unspecified acromioclavicular joint, sequela
S4351XA	S43.51XA	Sprain of right acromioclavicular joint, initial encounter
S4351XD	S43.51XD	Sprain of right acromioclavicular joint, subsequent encounter
S4351XS	S43.51XS	Sprain of right acromioclavicular joint, sequela
S4352XA	S43.52XA	Sprain of left acromioclavicular joint, initial encounter
S4352XD	S43.52XD	Sprain of left acromioclavicular joint, subsequent encounter
S4352XS	S43.52XS	Sprain of left acromioclavicular joint, sequela

TABLE SA.10: ICD Diagnosis Codes - Glenohumeral instability/hypermobility

ICD9 Code, WITHOUT decimals	ICD9 Code, WITH decimals	Description
NONE		

ICD10 Code WITHOUT decimals	ICD10 Code WITH decimals	Description
M25211	M25.211	Flail joint, right shoulder
M25212	M25.212	Flail joint, left shoulder
M25219	M25.219	Flail joint, unspecified shoulder
M25311	M25.311	Other instability, right shoulder
M25312	M25.312	Other instability, left shoulder
M25319	M25.319	Other instability, unspecified shoulder

TABLE SA.11 ICD Diagnosis Codes - Hypomobility/adhesive capsulitis

ICD9 Code, WITHOUT decimals	ICD9 Code, WITH decimals	Description
71841	718.41	Contracture of joint, shoulder region
71851	718.51	Ankylosis of joint, shoulder region
71951	719.51	Stiffness of joint, not elsewhere classified, shoulder region
7260	726.0	Adhesive capsulitis of shoulder**

ICD10 Code, WITHOUT decimals	ICD10 Code, WITH decimals	Description
M24511	M24.511	Contracture, right shoulder
M24512	M24.512	Contracture, left shoulder
M24519	M24.519	Contracture, unspecified shoulder
M24611	M24.611	Ankylosis, right shoulder
M24612	M24.612	Ankylosis, left shoulder
M24619	M24.619	Ankylosis, unspecified shoulder
M25611	M25.611	Stiffness of right shoulder, not elsewhere classified
M25612	M25.612	Stiffness of left shoulder, not elsewhere classified
M25619	M25.619	Stiffness of unspecified shoulder, not elsewhere classified
M7500	M75.00	Adhesive capsulitis of unspecified shoulder
M7501	M75.01	Adhesive capsulitis of right shoulder
M7502	M75.02	Adhesive capsulitis of left shoulder

TABLE SA.12 ICD Diagnosis Codes – Glenohumeral osteoarthritis

ICD9 Code WITHOUT decimals	ICD9 Code WITH decimals	Description
71511	715.11	Osteoarthrosis, localized, primary, shoulder region
71521	715.21	Osteoarthrosis, localized, secondary, shoulder region
71531	715.31	Osteoarthrosis, localized, not specified whether primary or secondary, shoulder region
71591	715.91	Osteoarthrosis, unspecified whether generalized or localized, shoulder region

ICD10 Code WITHOUT decimals	ICD10 Code WITH decimals	Description
M19011	M19.011	Primary osteoarthritis, right shoulder
M19012	M19.012	Primary osteoarthritis, left shoulder
M19019	M19.019	Primary osteoarthritis, unspecified shoulder
M19111	M19.111	Post-traumatic osteoarthritis, right shoulder
M19112	M19.112	Post-traumatic osteoarthritis, left shoulder
M19119	M19.119	Post-traumatic osteoarthritis, unspecified shoulder
M19211	M19.211	Secondary osteoarthritis, right shoulder
M19212	M19.212	Secondary osteoarthritis, left shoulder
M19219	M19.219	Secondary osteoarthritis, unspecified shoulder
M25711	M25.711	Osteophyte, right shoulder
M25712	M25.712	Osteophyte, left shoulder
M25719	M25.719	Osteophyte, unspecified shoulder

TABLE SA.13 ICD Diagnosis Codes – Non-specific shoulder disorders

ICD9 Code, WITHOUT/WITH decimals		Description
71941	719.41	Pain in joint, shoulder region**

ICD10 Code WITHOUT decimals	ICD10 Code WITH decimals	Description
M25511	M25.511	Pain in right shoulder
M25512	M25.512	Pain in left shoulder
M25519	M25.519	Pain in unspecified shoulder**
